# The Molecular Mechanism of Antioxidation of Huolisu Oral Liquid Based on Serum Analysis and Network Analysis

**DOI:** 10.3389/fphar.2021.710976

**Published:** 2021-11-01

**Authors:** Yihui Yin, Kai Zhang, Longyin Wei, Dongling Chen, Qian Chen, Mingjie Jiao, Xinxin Li, Jiaqi Huang, Zhexi Gong, Nianxin Kang, Fei Li

**Affiliations:** ^1^ School of Chinese Materia Medica, Beijing University of Chinese Medicine, Beijing, China; ^2^ School of Management, Beijing University of Chinese Medicine, Beijing, China

**Keywords:** serum pharmacochemistry, network pharmacology, huolisu oral liquid, antioxidation, molecular mechanism

## Abstract

Huolisu Oral Liquid (HLS), a well-known traditional Chinese medicine (TCM) prescription, is an over-the-counter drug that is registered and approved by the State Food and Drug Administration (Approval No. Z51020381). HLS has been widely applied in the clinical treatment of cognitive disorders and has effects on delaying aging. The antioxidant effects of HLS are closely related to its antiaging activities, but the underlying mechanisms are unclear. In this study, the potential antioxidant ingredients of HLS were screened based on serum pharmacochemistry and network pharmacology, and the potential mechanisms involved in HLS antioxidant effects were preliminarily explored. Further, the antioxidant effects of HLS were verified by *in vivo* and *in vitro* experiments. The results showed that potential antioxidant ingredients could affect the toxic advanced glycation end products-receptor for advanced glycation end products (TAGE-RAGE) signaling, mitogen-activated protein kinase (MAPK) signaling, interleukin (IL)-17 signaling, tumor necrosis factor (TNF) signaling, toll-like receptors (TLRs), cyclic adenosine monophosphate (cAMP) signaling, hypoxia-inducible factor (HIF)-1 signaling, and other related pathways by regulating GAPDH, AKT1, TP53, MAPK1, JUN, and other associated targets. Thus, HLS may reduce inflammation, control the release of inflammatory cytokines, and regulate mitochondrial autophagy and metabolic abnormalities to ultimately play an antioxidant role. This is the first study attempting to construct a multilevel network of “HLS-antioxidant targets” based on serum pharmacochemistry and network pharmacology to explore the relationship between HLS and antioxidation and the molecular mechanisms of antioxidation combined with bioinformatics functional analysis and lays a foundation for further elucidating the antioxidant mechanisms of HLS.

## Introduction

Huolisu Oral Liquid (HLS) is composed of *Reynoutria multiflora* (Thunb.), Moldenke (Polygonaceae; Polygoni multiflora radix praeparata), *Epimedium brevicornu* Maxim. [Berberidaceae; Epimedii folium (Yin Yanghuo)], *Polygonatum sibiricum* F. Delaroche (Asparagaceae; Polygonati rhizoma), *Lycium chinense* Mill. (Solanaceae; Lycii fructus), *Astragalus mongholicus* Bunge (Leguminosae; Astragali radix), and *Salvia miltiorrhiza* Bunge (Lamiaceae; Salviae miltiorrhizae radix et rhizoma) at a weight ratio of 50:15:22:15:22:11. Clinically, HLS is used to treat neurasthenia, depression, hair loss, insomnia, senile diabetic osteoporosis, and aging (Duan et al., 2020). According to the free radical theory of aging, the accumulation of free radicals causes biomolecular damage; leads to oxidative stress and damage to body tissues, which in turn induces diseases such as arteriosclerosis, inflammation, heart disease, and cancer; and accelerates human aging (Halliwell, 2009). Antiaging has become a scientific issue of worldwide concern, and finding suitable antiaging drugs is particularly important. Studies have shown that six constituents in HLS have antioxidant effects (Gao et al., 2012; Jiang et al., 2013; Ma et al., 2013; Liu et al., 2017; Ma et al., 2017; Xu and Lin, 2008). Yin Yanghuo can delay aging in via biological processes, such as antioxidation and anti-inflammatory processes, and may promote cell proliferation (Ma et al., 2017). *Reynoutria multiflora* (Thunb.) Moldenke can protect cardiomyocytes from oxidative damage by scavenging oxygen free radicals (Jiang et al., 2013). *Polygonatum sibiricum* F. Delaroche can enhance the body’s antioxidant capacity by inhibiting oxygen free radicals in aging animals (Zhu and Xu, 1999). There are many ingredients with complex structures and targeted activity in traditional Chinese medicine (TCM) compounds. The mechanism of the HLS activity is complex (Li, 2011; Yu and Lu, 2018; Sun et al., 2021); thus, it is particularly important to establish a new research model to clarify the mechanism of action of TCM constituent compounds.

Serum pharmacochemistry of TCM is based on traditional medicinal chemistry methods using modern separation and identification techniques such as liquid-phase and mass spectrometry to analyze and identify the components in the serum of experimental animals after oral administration of TCM (Wang et al., 2002). Serum medicinal chemistry of TCM takes the components that enter the blood at the entry point, reduces the interference of other components, and quickly determines the migration components in the serum (Wu et al., 2019). Ultra-high-performance liquid chromatography coupled with electrospray ionization hybrid linear trap quadrupole orbitrap high-resolution mass spectrometry (UPLC-LTQ-Orbitrap MS) can provide accurate relative molecular mass and multistage mass spectrometry information of a compound and can obtain a great deal of information about its structure, which enables to significantly improve the analysis and identification of chemical components of the complex prescription of TCM and the chemical components released into the serum. It has become an important means to study serum pharmaceutical chemistry of TCM (Kang et al., 2014).

Network pharmacology explores the relationship between drugs and diseases as a whole. Its principle is to construct a “components–targets–pathways” network and to study the relationship between components and targets and between targets and signal pathways. Through network pharmacology analysis, we screened out the targets and signaling pathways of the TCM compound and combined with existing research to explain the mechanism of the compound. Its holistic and systemic nature is consistent with the features of compound prescription of TCM in treating diseases. Therefore, network pharmacology has unique advantages in the research of compound prescription of TCM. Analysis from the perspective of network pharmacology is helpful to comprehensively identify the network regulatory effects of TCM on the body and to explore the mechanism of action of compound prescriptions on the body from the cell to the molecular level (Luo et al., 2020). The molecular docking technology can elucidate the mechanisms of action between active ingredients and target proteins at the molecular level, which is widely used in virtual screening of effective substances in TCM, and contributes to the preliminary clarification of the mechanism of action of TCM in treating diseases (Chen et al., 2005).

*Caenorhabditis elegans* (*C. elegans*) has a characteristic short growth cycle, a fast reproduction cycle, and high homology with human genes. It is a useful model for studying antioxidant effects of HLS (Sulston et al., 1983; Hunter et al., 2010; Jin et al., 2020).

**FIGURE 1:**
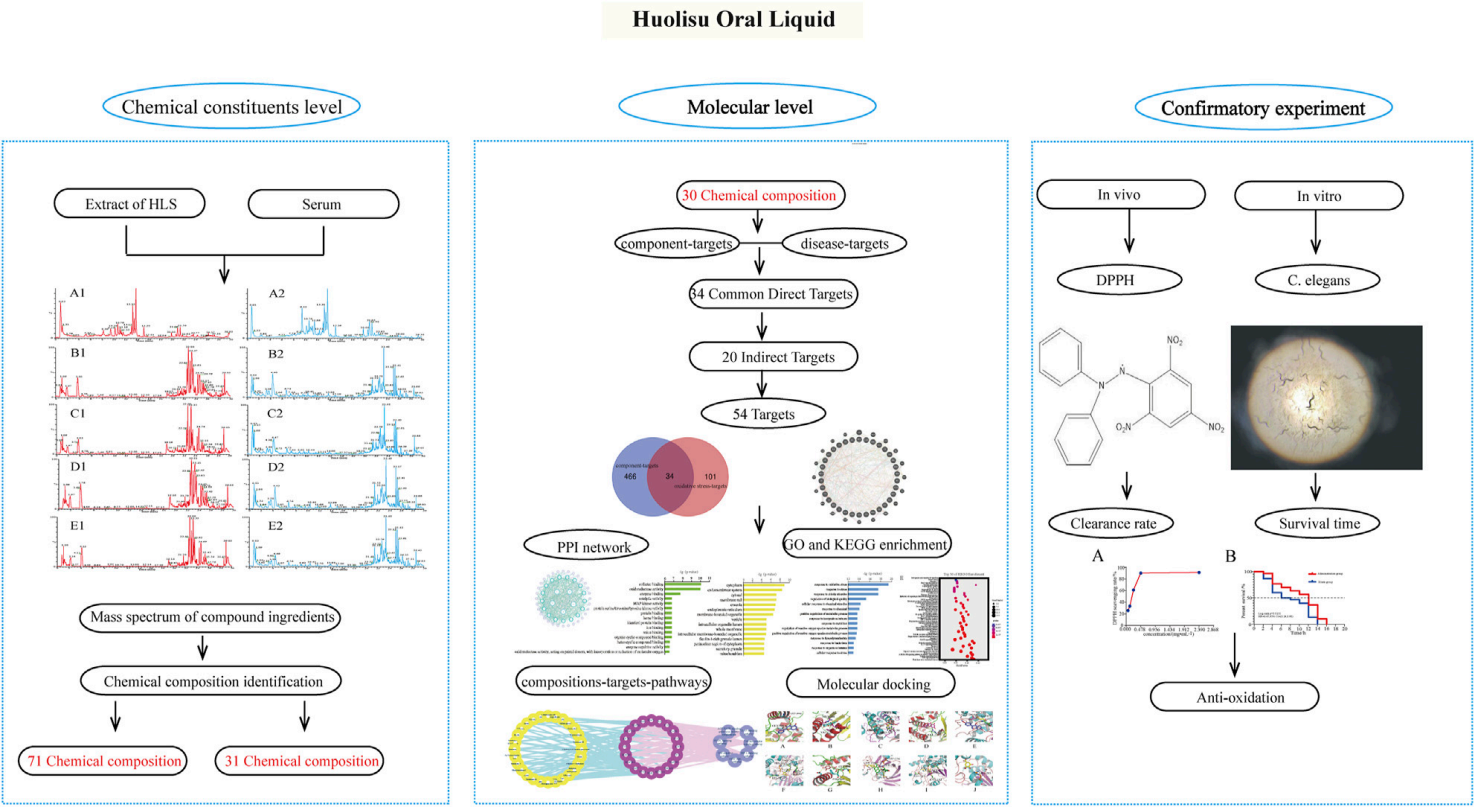
Framework of the antioxidant effects of HLS.

In this study, the components of HLS entering the blood circulation were analyzed by serum pharmacochemistry, and the transmission of chemical components from the compound prescription of TCM to the body was determined. Based on network pharmacology, we screened the active components of HLS having antioxidant activity present in the blood and analyzed their potential targets, biological processes, and signaling pathways. The antioxidant activity of HLS at the molecular level was explored, and the correlation between chemical components of HLS and clinical efficacy was clarified. Finally, we verified the antioxidant activity of HLS through a series of related experiments. Figure 1 shows the framework of our study.

## Materials and Methods

### Materials and Reagents

HLS was obtained from Chengdu Diao Group Tianfu Pharmaceutical Co., Ltd. (Chengdu, China). HLS’s procedure is shown in Supplementary Material S1. As reference standards, eight pure compounds were used (purity ≥98%): epimedin A, epimedin B, epimedin C, emodin, tanshinone ΙΙA, cryptotanshinone, gallic acid, and icariin. All were purchased from Shanghai Shidande Standard Technology Service Co., Ltd. (Shanghai, China). The juglone standard (purity ≥98%) was purchased from Shanghai Yuanye Biotechnology Co., Ltd. (Shanghai, China). Wild-type *C. elegans* N2 and *Escherichia coli* OP50 (*E. coli* OP50) were donated by the Genetics and Developmental Biology Department of the Chinese Academy of Sciences (Beijing, China). MS-grade acetonitrile, methanol, and formic acid were purchased from Thermo Fisher Scientific (Waltham, MA, USA). Distilled water was obtained from Watsons (Shenzhen, China). Analysis-grade anhydrous ethanol was purchased from Beijing Chemical Plant (Beijing, China). Agar and peptone purchased from Beijing Aobostar Biotechnology Co., Ltd. (Beijing, China). 1,1-Diphenyl-2-picrylhydrazyl (DPPH; purity ≥96%) was purchased from Shanghai Yuanye Biotechnology Co., Ltd. (Shanghai, China). Cholesterol (purity ≥99%) was purchased from Beijing Bootota Technology Co., Ltd. (Beijing, China). Sodium chloride (purity ≥99%), calcium chloride (purity ≥96%), and magnesium sulfate (purity ≥99%) were purchased from Beijing Reagent Co., Ltd. (Beijing, China).

### Analysis of Components of HLS Based on UPLC-LTQ-Orbitrap MS

#### Preparation of Sample Solutions

A total of 2 ml of HLS was precisely weighed and placed in a 50 ml volumetric flask. A solution of 70% methanol was added to the 50 ml volume mark, and the flask was weighed. After ultrasonic treatment for 15 min, the flask was removed and cooled to room temperature. After weighing, additional 70% methanol was added to supplement the weight loss, and the flask was shaken vigorously to obtain a test solution of 0.01 g/mL^−1^. The contents of the volume bottle were filtered through a 0.22 μm microporous membrane.

#### Preparation of Standards

For the mixed solution, 1 mg each of epimedin A, epimedin B, epimedin C, epinephrine, tanshinone IIA, cryptotanshinone, gallic acid, and icariin was weighed and placed in a 10 ml volumetric flask and filled with 70% methanol to the 10 ml volume. The solution was subjected to ultrasound at room temperature for 10 min to allow dissolution. The solution was diluted to obtain a mixed standard solution of 0.1 mg/mL^−1^. The contents of the volumetric flask were filtered through a 0.22 μm microporous membrane.

### Study of the Serum Pharmacochemistry of Antioxidation of HLS

#### Mice Models

A total of 28 SPF KM female mice were purchased from Sbeford Biotechnology Co., Ltd. (Beijing, China), with license number SCXK (Beijing) 2019-0010. At the time of the experiment, the mice were 10 weeks old and weighed 28 ± 2 g. They were raised with free access to drinking water and standard laboratory mice chow at the Animal Experimental Center of Liangxiang Campus of Beijing University of TCM (temperature: 23 ± 2°C, humidity: 35 ± 5%, illumination cycle: 12 h day–night alternation). The mice were randomly divided into 2 groups with 14 mice in each group. One group was given HLS, which was the treatment group; the other group was given distilled water, which was the blank group. The treatment regimen is shown in Table 1. All experimental procedures were conducted in accordance with Chinese national laws and local guidelines. The animal experiment was approved by the Animal Ethics Committee of Beijing University of TCM (BUCM-4-2020092903-3117).

**TABLE 1:** Regimen of intragastric administration in mice.

Grouping	Number	Drug	Dose	Method of administration	Exposure period
Control group	14	Distilled water	33 ml/kg^−1^/d^−1^	Intragastric administration	7 days
Treatment group	14	HLS	33 ml/kg^−1^/d^−1^	Intragastric administration	7 days

#### Collection and Pretreatment of Mouse Samples

According to the treatment plan, mice in each group were given the corresponding treatments for 7 days. On the 7th day, the two groups of mice were first fasted for 12 h and then were given the corresponding treatments. Mice in each group were subdivided evenly into two smaller groups: in one, orbital blood was collected after 30 min, and the other, collection occurred 60 min after administration. The blood samples were placed in a centrifuge tube, and after standing for 30 min, the serum was centrifuged at 15,000 r/min^−1^ for 15 min and then centrifuged at 15,000 r/min^−1^ for 10 min. The supernatant was collected and stored at −80°C.

#### Treatment of Serum Samples

The serum samples obtained at 30 and 60 min after treatment were mixed in equal amounts. A 1200 μL volume of acetonitrile was added to the 400 μL total volume of the mixed serum sample. The mixture was ultrasonically treated in an ice water bath for 10 min and vortexed for 1 min. The mixture was centrifuged at 4°C for 15 min at 12,000 rpm, and the supernatant was collected and dried under nitrogen. The dry matter was dissolved in 200 or 100 μL of 70% methanol (enriched 2 and 4 times), and then, the solutions were centrifuged at 4°C for 15 min at 12,000 rpm. The supernatant was analyzed by UPLC-LTQ-Orbitrap MS.

### Qualitative Analysis of Chemical Components of HLS and Drug-Containing Serum

#### Chromatographic Conditions

Chromatographic column: Waters Acquity UPLC BEH-C18 (2.1 × 100 mm, 1.7 μm; Waters Corporation, Milford, MA, USA); mobile phase A was 0.1% formic acid water, and mobile phase B was acetonitrile. Elution program: 0–4 min, 95%–90% A; 4–9 min, 90%–75% A; 9–12 min, 75%–70% A; 12–17 min, 70%–69% A; 17–25 min, 69%–0% A; 25–27 min, 0% A; 27–28 min, 0%–95% A; 28–30 min, 95% A; flow rate: 0.3 ml min^−1^; sample volume: 5 μL; column temperature: 30°C.

#### Conditions for Mass Spectrometry

A heated electrospray ionization (HESI) ion source was used to scan in the positive ion mode and the negative ion mode. The scanning range was m/z 50–2,000 Da, and the ion source temperature was 350°C. The ionization source voltage, capillary voltage, and tube lens voltage were 4 kV, 35 V, and 110 V, respectively. The sheath gas flow rate was 40 arb, and the auxiliary gas flow rate was 20 arb; both were nitrogen sources. The data scanning resolution of the primary MS was 30,000 using the Fourier transform high-resolution scanning mode. Secondary and tertiary mass spectrometry data were obtained by dependency scanning and decomposed by collision-induced dissociation.

#### Data Processing

A database of the chemical constituents of HLS was established by consulting the literature and collecting chemical constituents’ information of single botanical drugs. The Xcalibur 4.2 workstation was used to analyze data. Based on the accurate relative molecular mass, quasi-molecular ion peaks, multilevel ion fragments, and other data obtained from mass spectrometry and a quality control deviation range of δ ≤ 10 × 10^−6^, we identified the chemical composition of HLS. By comparing the chemical components in HLS and serum, the chemical components absorbed in mouse serum were identified. The components entering the blood have a designated chemical structure and Pubchem CID, and some are quantitatively recorded in each botanical drug in the Chinese Pharmacopoeia (2020) (Committee 2020).

### Network Pharmacology and Molecular Docking Study on the Antioxidant Mechanism of HLS

#### Construction of the Blood Ingredient Bank of HLS

We collected the chemical components in the mice serum identified under the conditions described in section 2.4.3.3 to construct the HLS blood component database.

#### Acquisition of “Component Targets”

Based on the blood components identified in HLS described, we searched the Traditional Chinese Medicine Systems Pharmacology (TCMSP) (https://tcmspw.com) database with the Chemical name to obtain the target molecules. Each blood component was queried in the PubChem (https://pubchem.ncbi.nlm.nih.gov/) database to predict potential targets, and the Canonical SMILES of HLS blood components were downloaded. In the Swiss Target Prediction (http://www.swisstargetprediction.ch) database, we selected a species, *Homo sapiens*, and entered the Canonical SMILES of the blood components to obtain the corresponding target and common name. Based on the UniProt (https://www.uniprot.org) database, we searched “UniProtKB” to convert target names and common names into gene names. The organism was selected as Human.

#### Acquisition of “Disease Targets”

We used “oxidative stress” and “anti-oxidant” as keywords and used the PubMed (https://pubmed.ncbi.nlm.nih.gov) database to comprehensively search all MeSH terms. We searched for genes related to antioxidants using the GeneCards (https://www.genecards.org) database and selected genes with a score >20.

#### Identification of Common Targets

We import the targets corresponding to the blood components of HLS and the disease targets of antioxidants into the Draw Venn Diagram online mapping tool to obtain the components–diseases intersection targets and the Venn diagram of the two. The intersection targets were the common targets. The common targets were defined as the direct antioxidant targets of HLS.

#### Acquisition of Indirect Targets

The direct antioxidant targets of HLS were imported into the GeneMANIA (https://genemania.org/) database under the condition of “*Homo sapiens*” to obtain targets having an indirect relationship with the antioxidant effects of HLS.

#### Construction and Analysis of the Protein–Protein Interaction Network

We used the String (https://www.string-db.org/) database and imported the direct and indirect targets of the antioxidant effects of HLS as “Multiple Proteins by Names/Identifiers,” for protein–protein interaction (PPI) analysis and limited the species to *Homo sapiens*. Then, we imported the above-constructed PPI network information into Cytoscape 3.7.2 software and used the Network Analyzer function to analyze the topology of the PPI network to predict and visualize the important protein nodes and subnets in the network.

#### Enrichment Analysis Using Gene Ontology and Kyoto Encyclopedia of Genes and Genomes Pathways

We introduced the targets of the HLS antioxidant mechanism into String to obtain the files that could be used to analyze the biological process, cellular components, molecular functions, and pathways of these genes. GraphPad-Prism 9.0.2 was used to visualize the “enrichment. Process,” “enrichment. component,” and “enrichment. Function,” and we used Omicshare (http://www.omicshare.Com/tools/index.Php/) to visualize “enrichment. KEGG.”

#### Molecular Docking of Target Proteins With Their Reverse-Screened Chemical Components

We downloaded the three-dimensional (3D) structures of HLS active ingredients (ligands) in the CDX format through PubChem. The energy of ligands was minimized by ChemBio 3D Ultra 14.0 software and saved as pdbqt files for the Autodock Tools software. We used the Protein Data Bank (PDB) (http://www.rcsb.org/) database to download the PDB format files of 3D structures of the target proteins (receptors) and used AutoDock Tools 1.5.6 software to hydrogenate the receptors and save them as the pdbqt files. The ligands and receptors were docked by AutoDock Vina software. The molecular docking results use the binding free energy as the standard to evaluate the binding of the receptor protein and the compound. The lower the binding energy, the more stable the conformation ligand binding, the greater the probability of binding, and the more reliable were the docking results. In general, if the binding energy of the compound molecule to the receptor was less than −5.0 kcal mol^−1^, it had better binding activity, and if the binding energy between the compound molecule and the receptor was less than −7.0 kcal mol^−1^, it had strong binding activity (Hsin et al., 2013).

### Study on Antioxidation of HLS *in vivo*

#### Preparation of the *C. elegans* Feed

A volume of 4.9 ml of *E. coli* OP50 suspension and 100 μL of HLS were combined and mixed. Also, 100 μL of this solution was used as a feed for *C. elegans* in the administration group. A 100 μL volume of *E. coli* OP50 suspension was used as a feed for the blank group of *C. elegans*.

#### Preparation of the Culture Medium

Preparation of the NGM medium: We added 0.6 g of NaCl, 0.5 g of peptone, 3.4 g of agar powder, and a small amount of cholesterol to the conical flask, and then, all powders were dissolved in 200 ml of distilled water. The solution was sterilized at 121°C and 0.12 MPa for 20 min and then cooled to 55°C at room temperature. Finally, 100 μL of 1 mol/L^−1^ CaCl_2_, 100 μL of 1 mol/L^−1^ MgSO_4_, and 5 ml of 1 mol/L^−1^ phosphate-buffered saline (PBS) buffer were added and mixed well, and the solution was poured into a Petri dish.

#### Culture and Synchronization of *C. elegans*

The *C. elegans* were cultured at 20°C in NGM dishes containing OP50. Under sterile conditions, 40–50 adults at the oviposition stage were picked and cultured in the NGM medium, and all adults were picked out after oviposition for 2 h for synchronization of *C. elegans*. *E. coli* OP50 was dropped onto the oviposition plate, and the eggs were cultured in a sterile biochemical incubator at 20°C for 12 h to obtain simultaneous *C. elegans* cultures at the L1 stage.

#### Oxidative Stress Studies

The experiment was divided into a blank group (2 μL *E. coli* OP50) and a treatment group (2 μL HLS). Several synchronized L1 larvae were randomly selected and transferred to each of the two culture mediums indicated above, and larvae were developed at 20°C until the adult stage. A total of 30 adult worms from each group were transferred to a medium containing 400 μmol/L^−1^ juglone to establish oxidative damage models. We counted the number of surviving *C. elegans* every 2 h to observe any increase in the survival rate of *C. elegans* under oxidative stress. Each group of experiments was measured three times in parallel. The average values of the two groups of data were statistically analyzed by Graphpad Prism 8.0 software. A *p-value* < 0.05 indicated that differences were statistically significant.

### Study of the Antioxidative Activity of HLS *In Vitro*

#### Preparation of the DPPH Solution

The DPPH solution was prepared by weighing 3.94 mg of DPPH in a 100 mL brown volumetric flask with anhydrous ethanol.

#### Preparation of the Sample Solution

HLS was first lyophilized, and then absolute ethanol was added to the powder obtained to prepare solutions of different concentrations: 0.059 mg/mL^−1^, 0.119 mg/mL^−1^, 0.239 mg/mL^−1^, 0.478 mg/mL^−1^, and 2.390 mg/mL^−1^.

#### Scavenging Ability of HLS to DPPH

The absorbance values were measured as indicated in Table 2, and each sample was measured three times in parallel to obtain the average value.

**TABLE 2:** Sample preparation.

Sample	Content
A_0_	0.1 ml of DPPH solution + 0.1 ml of absolute ethyl alcohol
A_i_	0.1 ml of DPPH solution + 0.1 ml of the sample solution
A_j_	0.1 ml of the sample solution + 0.1 ml of absolute ethyl alcohol

Free radical scavenging rate (%) = $1\;–\;\frac{Ai\;–\;Aj}{A0}\times 100\%.$

## Results

### Serum Pharmacochemical Analysis of the Antioxidant Activity of HLS

#### Characteristics of Chemical Components in HLS

**FIGURE 2:**
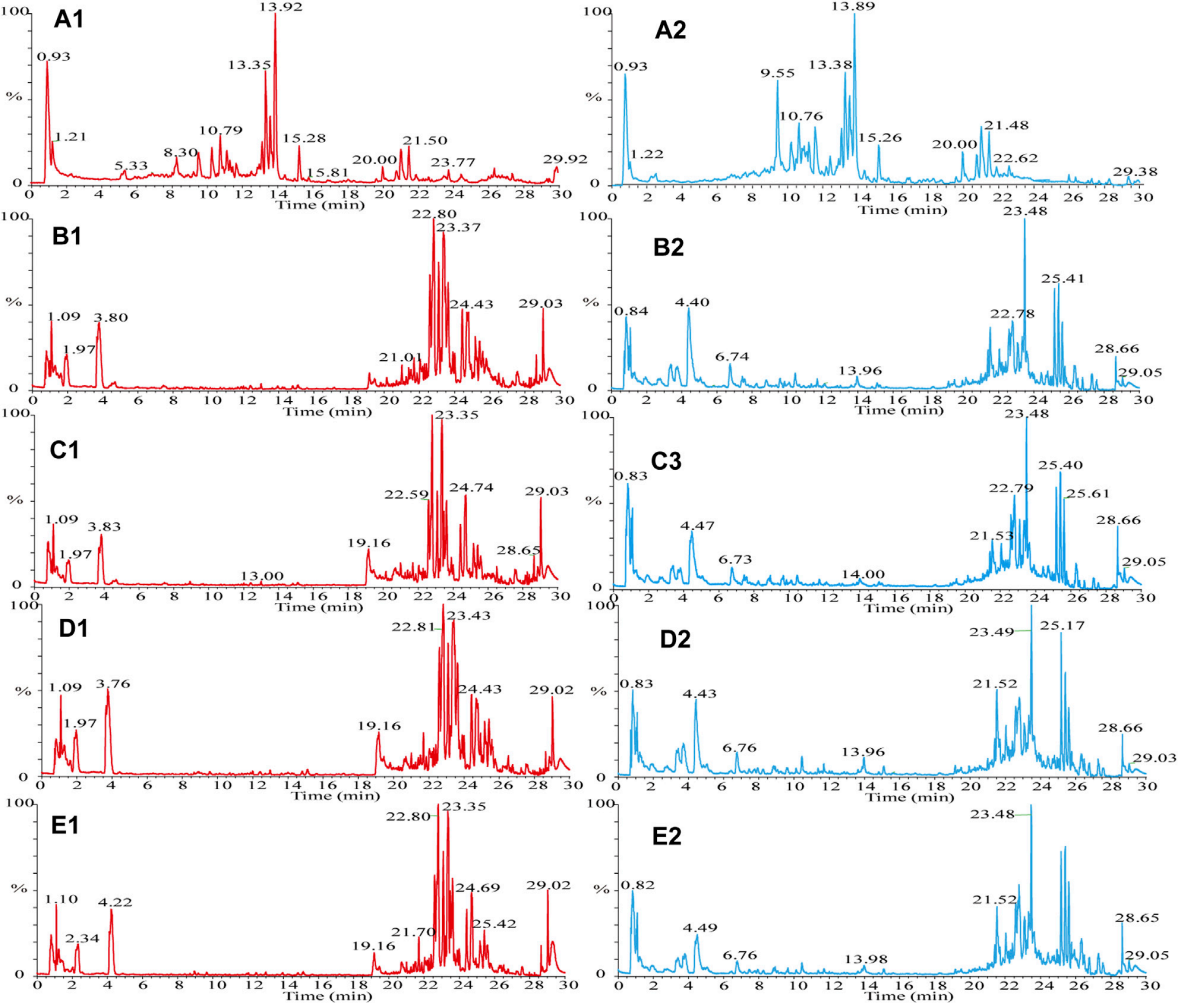
TIC diagram. **(A)** HLS, **(B–E)** mouse serum, (A1) HLS (HESI^+^), (A2) HLS (HESI^−^), (B1) drug group enriched 4 times (HESI^+^), (B2) drug group enriched 4 times (HESI^−^), (C1) drug group enriched 2 times (HESI^+^), (C2) drug group enriched 2 times (HESI^−^), (D1) blank group enriched 4 times (HESI^+^), (D2) blank group enriched 4 times (HESI^−^), (E1) blank group enriched 2 times (HESI^+^), and (E2) blank group enriched 2 times (HESI^−^).

**FIGURE 3:**
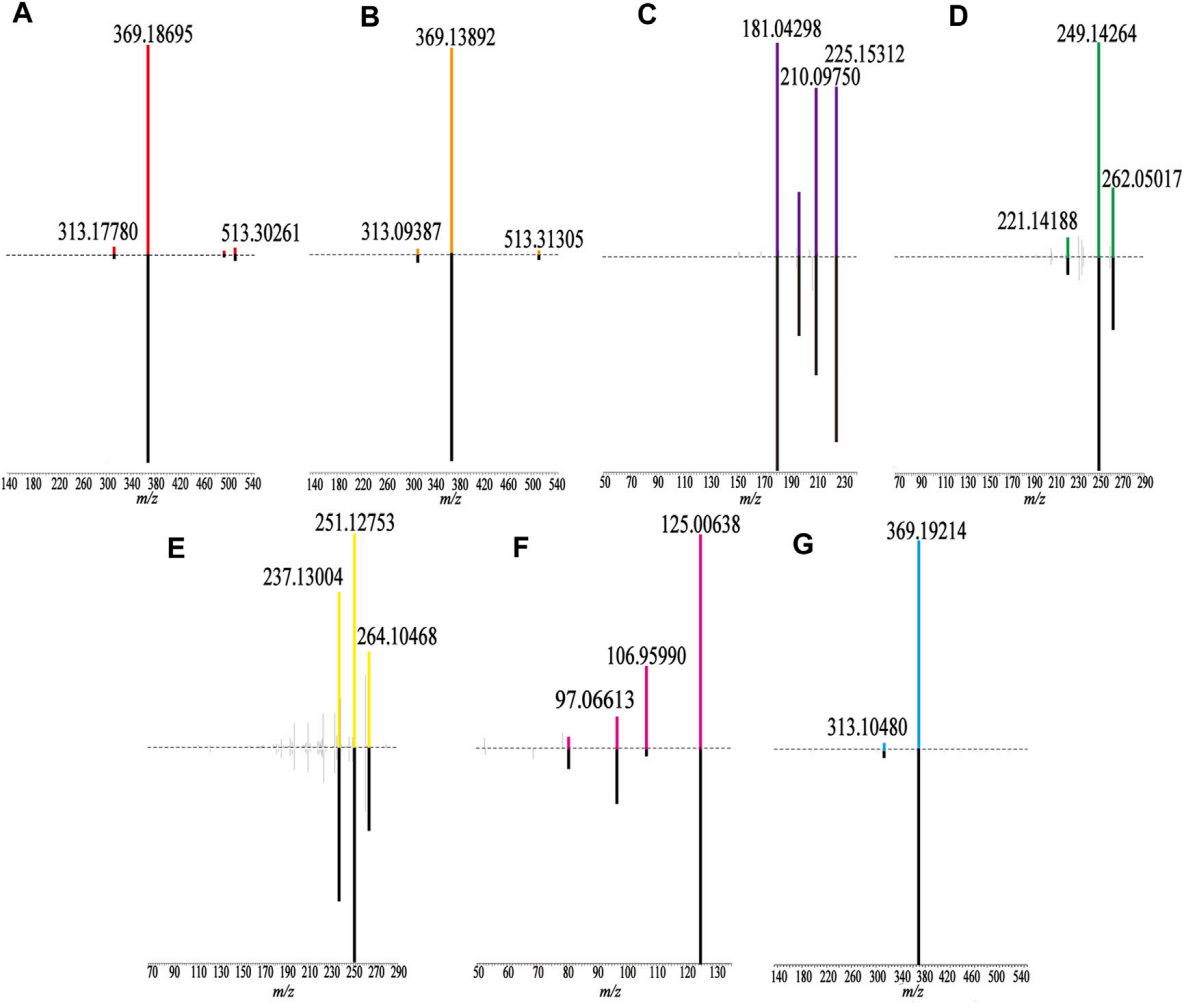
Comparison of fragment ions (MS^3^) between the sample (up) and the reference substance (down). **(A)** Epimedin A, **(B)** Epimedin B, **(C)** Emodin, **(D)** Tanshinone IIA, **(E)** Cryptotanshinone, **(F)** Gallic acid, and **(G)** Icariin.

The samples were scanned in the positive and negative ion modes to obtain the total ion chromatograms (TIC) diagram of the test solution of HLS shown in Figure 2A. The three-stage mass spectrometry information of seven chemical components identified from HLS was consistent with that of the reference substances, and their mirror images are shown in Figure 3. A total of 71 chemical components in HLS were identified in this study. Among them were 32 flavonoids and 9 phenolic acids. In addition, other classes of molecules were identified, such as diterpenoids, anthraquinones, and glycosides. The details are shown in Table 3 **.**

**TABLE 3:** Chemical components of HLS and mouse serum identified by LTQ-orbitrap MS.

No	Rt (min)	Formula	Ions	Calculated *m/z*	Observed *m/z*	Error (*ppm*)	Identify	MS^2^	MS^3^	Category	From
1	0.87	C_12_H_22_O_11_	[M-H]^-^	341.10893	341.10938	4.521	Sucrose	179.03477	88.81845	Saccharides	H
								160.97990	143.02516, 161.01266		
								142.94624			
2	0.93	C_5_H_11_NO_2_	[M + H]^+^	118.08625	118.08594	−2.669	Betaine	118.97800	/	Alkaloids	H,S
3	1.21	C_5_H_7_NO_3_	[M + H]^+^	130.04986	130.04945	−3.277	L-pyroglutamic acid	83.88052	83.91258	Others	H
								111.84616			
4	1.31	C_10_H_13_N_5_O_4_	[M + H]^+^	268.10403	268.10355	−1.792	Adenosine	135.93582	136.00859	Others	H,S
5	1.64	C_7_H_6_O_5_	[M-H]^-^	169.01424	169.01486	10.119	Gallic acid	124.83292,	125.00638	Tannins	H,S
								150.89281,	106.95990		
								106.97633	97.06613		
									78.93007		
6	2.68	C_9_H_8_O_4_	[M-H]^-^	179.03498	179.03560	9.578	Caffeic acid	134.91914	134.96643	Phenylpropanoids	H,S
									107.02103		
7	3.26	C_6_H_6_O_3_	[M + H]^+^	127.03897	127.03860	−2.917	5-Hydroxymethylfurfural	126.93042	/	Others	H,S
								128.00446			
8	4.44	C_11_H_12_N_2_O_2_	[M + H]^+^	205.09715	205.09665	−2.458	L-Tryptophan	187.94771	145.93520	Others	H,S
								177.10249	144.02914, 142.02914		
9	6.22	C_16_H_18_O_9_	[M-H]^-^	353.08780	353.08853	5.159	Chlorogenic acid	172.98177	/	Phenolic acids	H,S
								179.04092			
								191.02545			
10	7.17	C_26_H_32_O_14_	[M-H]^-^	567.17192	567.17358	4.845	Tetrahydroxystilbene-O-dihexoside	447.14557	285.07687	Glycosides	H
								477.27240	267.14404		
								549.28888			
								405.17401			
								243.09369			
11	7.35	C_16_H_18_O_8_	[M-H]^-^	337.09289	337.09369	5.625	5-p-coumaroylquinic acid or 3-O-p-coumaroylquinic acid	190.98824,	126.98746	Phenolic acids	H,S
								162.99219	84.92613		
									173.02827		
12	8.30	C_20_H_23_NO_4_	[M + H]^+^	342.16998	342.16916	-2.410	Magnolflorine	297.13049	265.03793	Alkaloids	H,S
								265.11176	282.07861		
								311.14453			
13	8.30	C_18_H_16_O_4_	[M + H]^+^	297.11213	297.11157	−1.907	Danshenol B	265.05536	237.08357	Diterpenoids	H
								282.09186	219.00456		
								237.08841	207.03113		
14	8.51	C_21_H_22_O_9_	[M-H]^-^	417.11910	417.12064	6.308	Cassialoin	255.12778	/	Anthraquinones	H
								297.02570			
								279.15808			
15	8.99	C_27_H_30_O_15_	[M-H]^-^	593.15119	593.15302	4.929	Quercetin-3,7-dirhamnoside	447.18115	301.08011	Flavonoids	H,S
								301.12161			
16	9.26	C_22_H_22_O_10_	[M + H]^+^	447.12857	447.12720	−3.071	Calycosin-7-O-β-D-glycoside	285.07806	/	Flavonoids	H
17	9.28	C_33_H_40_O_19_	[M-H]^-^	739.20910	739.21161	4.877	Kaempferol-3-O-rhamnose-glucose-7-O-rhamnoside	593.22723	431.26678	Flavonoids	H
								429.65814, 431.27588			
18	9.55	C_20_H_22_O_9_	[M-H]^-^	405.11910	405.12042	5.952	Stilbene glucoside	243.03439	148.88429	Glycosides	H,S
								225.07831	225.00101		
								173.08704	136.92899		
								137.08725			
19	9.61	C_27_H_30_O_14_	[M-H]^-^	577.15627	577.15784	4.606	Kaempferitrin	431.09601	285.06454	Flavonoids	H
								285.03131			
20	9.78	C_27_H_22_O_12_	[M-H]^-^	537.10384	537.10565	5.395	Lithospermic acid or its isomers	493.19452	295.06110	Phenolic acids	H
								295.08038	313.12189		
								313.18640	383.18323		
								383.14441			
								519.25909			
21	10.25	C_51_H_82_O_25_	[M-H]^-^	1093.50724	1093.51086	4.313	Kingianoside E or its isomers	931.50391	751.52606	Steroidal saponins	H
								769.47546	769.44641		
								751.57208	913.47827		
								913.44263			
22	10.32	C_9_H_10_O_5_	[M-H]^-^	197.04554	197.04628	9.288	Danshensu	178.93611	134.95024	Phenolic acids	H,S
								153.03618			
								135.04256			
								122.96371			
								108.89146			
								72.83027			
23	10.42	C_27_H_26_O_13_	[M-H]^-^	557.13006	557.13208	3.113	Tetrahydroxystilbene-O-(galloyl)-hexoside	313.15710	168.91565	Glycosides	H
								243.00484	124.97063		
								405.22217			
								169.07069			
24	10.64	C_17_H_16_O_5_	[M + H]^+^	301.10705	301.10663	−1.395	3-Hydroxy-9,10-dimethoxypterocarpan	283.03949	265.04443	Flavonoids	H,S
								257.18552	237.03067		
								286.08417	176.06126		
25	10.94	C_38_H_48_O_20_	[M-H]^-^	823.26661	823.27020	5.685	Diphylloside A or its isomers	661.23029	352.28168	Flavonoids	H
								353.27780	353.15125		
									481.21490		
26	11.23	C_38_H_48_O_19_	[M-H]^-^	807.27170	807.27478	5.171	Diphylloside B	645.27783	352.17627	Flavonoids	H
								514.27106	481.30072		
								481.23987			
								353.27399, 351.25790			
27	11.01	C_18_H_16_O_8_	[M-H]^-^	359.07724	359.07834	6.116	Rosmarinic acid	160.92877	132.93008,160.94527	Phenolic acids	H
								178.95465			
								197.04767			
								223.09552			
28	11.05	C_37_H_46_O_19_	[M-H]^-^	793.25605	793.25958	5.830	Icariin E	631.33746	352.09851	Flavonoids	H
									481.19055		
									499.30707		
29	11.31	C_32_H_38_O_15_	[M-H]^-^	661.21379	661.21637	5.555	Epimedoside A	353.14063	298.0885	Flavonoids	H
								499.28992			
								395.18359			
								515.26422			
30	11.66	C_36_H_30_O_16_	[M-H]^-^	717.14610	717.14850	4.865	Salvianolic acid B or its isomers	519.19672	321.06061	Phenolic acids	H
								321.07117	339.10473		
								673.22491	279.13696		
								537.06085			
31	12.32	C_39_H_48_O_20_	[M-H]^-^	835.26661	835.26941	4.657	Demethylanhydroicaritin-7-O-glucopyranosyl-3-O-acetylated rhamnopyranosyl-xylopyranoside	673.27332	353.17456	Flavonoids	H
								353.18744	255.03459		
32	12.46	C_32_H_38_O_16_	[M + H]^+^	679.22326	679.22119	-3.050	Hexandraside E	355.15363	299.07562	Flavonoids	H
								517.16699			
								299.14502			
33	12.49	C_26_H_22_O_10_	[M-H]^-^	493.11402	493.11624	6.726	Salvianolic acid	295.04993	158.93649	Phenolic acids	H,S
								313.04919	277.02839		
								383.12494	108.95323		
								203.05615			
34	12.64	C_39_H_50_O_21_	[M + H]^+^	839.29682	839.29523	−1.895	Epimedin AΙ	531.18176	369.21112	Flavonoids	H
								677.30505			
								369.19308			
35	12.88	C_45_H_74_O_19_	[M-H]^-^	917.47515	917.47778	4.059	(3β,25R)-26-(β-D-glucopyranosyloxy)-22-hydroxyfurost-5-en-3-yl4-O-β-D-glucopyranosyl-β-D-galactopyranoside	755.52722	593.47650	Steroidal saponins	H
								593.44800	413.51624		
									737.56909		
36	12.92	C_16_H_12_O_5_	[M-H]^-^	283.06119	283.06219	7.384	Calycosin	268.02307	239.95195	Flavonoids	H,S
									210.97505		
									224.03589		
37	13.17	C_39_H_50_O_20_	[M + H]^+^	839.29682	839.29480	−2.407	Epimedin A	531.21881	369.18695	Flavonoids	H
								369.23535			
								677.15875			
38	13.49	C_26_H_20_O_10_	[M-H]^-^	491.09837	491.10071	6.998	Salvianolic acid C	293.11026	265.08508	Phenolic acids	H
								311.06104	249.04556		
								249.08069	276.09039		
								265.04987			
39	13.35	C_38_H_48_O_19_	[M + H]^+^	809.28625	809.28430	−2.416	Epimedin B	659.29468	369.13892	Flavonoids	H,S
								409.22931			
40	13.61	C_39_H_50_O_19_	[M-H]^-^	821.28735	821.29095	5.716	Epimedin C	658.32880	353.04584	Flavonoids	H
								368.23706	310.08832		
								367.21384			
								659.22528			
41	13.73	C_21_H_20_O_10_	[M-H]^-^	431.09837	431.10022	6.836	Torachrysone 8-O-glucoside	269.06149	225.02304	Glycosides	H,S
								311.12726	241.02071		
								293.08362	269.08868		
42	13.89	C_33_H_40_O_15_	[M + H]^+^	677.24399	677. 24164	−3.480	Icariin	531.14288	531.14288	Flavonoids	H,S
								369.21533	369.21533		
								313.08530	313.08530		
43	13.89	C_27_H_30_O_11_	[M-H]^-^	529.17153	529.17255	3.991	Icariside Ι	382.12506	/	Flavonoids	H
								367.26300			
44	14.38	C_26_H_28_O_11_	[M-H]^-^	515.15588	515.15826	6.740	Icariin C	353.13477	298.15164	Flavonoids	H
								309.19391	297.10327		
								219.12825			
								325.17279			
								297.15009			
45	14.64	C_39_H_48_O_21_	[M-H]^-^	851.26153	851.26440	4.658	Anhydroicaritin-3-o-rhamnopyranosyl-glucuronic	513.26160	366.15967	Flavonoids	H
								689.30646			
								555.27051			
46	15.09	C_17_H_16_O_6_	[M + H]^+^	317.10196	317.10144	−1.665	Dihydroxy-trimethoxy DHIF	134.93385	/	Flavonoids	H
								289.12463			
								299.17441			
								163.03706			
47	15.26	C_39_H_48_O_19_	[M-H]^-^	819.27170	819.27441	4.644	Anhydroicaritin-3-O-rhamnopyranosyl(1-4)-furan acid-7-O-glucopyranoside	367.13501	352.12796	Flavonoids	H
								657.24683	309.13480		
								639.42072	297.08844		
								529.30988			
								409.19366			
48	15.28	C_39_H_48_O_19_	[M + H]^+^	821.28625	821.28461	−2.004	3‴-Carbonyl-2″-β-L-quinovosyl-icariin	677.31866	/	Flavonoids	H
								531.23340			
								369.20304			
								313.06561			
49	15.64	C_15_H_10_O_6_	[M-H]^-^	285.04046	285.04169	8.159	ω-Hydroxyemodin	241.00211	/	Anthraquinones	H,S
								257.02148			
								285.14716			
								211.09290			
								224.05875			
								268.14087			
50	15.64	C_15_H_10_O_6_	[M-H]^-^	285.04046	285.04169	8.159	Kaempferol	241.00211	/	Flavonoids	H,S
								257.02148			
								285.14716			
51	16.86	C_23_H_22_O_11_	[M-H]^-^	473.10893	473.11102	6.726	Emodin-8-O-(6′-O-acetyl)-β-D-glucopyranoside	268.96448	225.07561	Anthraquinones	H
								311.11444	241.01837		
								293.09479	269.01453		
								225.02789			
52	16.86	C_23_H_22_O_11_	[M-H]^-^	473.10893	473.11102	6.726	Acetylemodin-O-hexor emodin-O-(acetyl)-hexoside	269.07031	225.07561	Anthraquinones	H
								311.09891,292.99927	241.01837		
								225.13992	269.01453		
								240.27361			
53	16.79	C_27_H_22_O_12_	[M-H]^-^	537.10384	537.10583	5.730	Salvianolic acid H/I/J	493.19452	/	Phenolic acids	H
								313.18640			
								339.13208			
								295.08038			
54	18.07	C_16_H_12_O_4_	[M-H]^-^	267.06628	267.06726	7.768	Formononetin	252.00014	222.96834	Flavonoids	H,S
									208.02678		
									195.06418		
55	18.36	C_31_H_36_O_14_	[M + H]^+^	633.21778	633.21631	−2.325	Ikarisoside F	501.16180	465.14722	Flavonoids	H
								483.12378	483.25656		
								465.22064	397.20908		
56	19.60	C_46_H_74_O_19_	[M-H]^-^	929.47515	929.47864	4.932	(3β,25R)-furost-5-en-12-one,3-[(6-deoxy-4-O-β-D-glucopyranosyl-β-D-galactopyranosyl)oxy]-26-(β-D-glucopyranosyloxy)-22-methoxy	911.24689	/	Steroidal saponins	H
								737.60858			
								893.11871			
57	19.64	C_26_H_28_O_10_	[M-H]^-^	499.16097	499.16293	6.123	Icariside A	352.27484	297.00586	Flavonoids	H
								353.15775	309.11832		
									284.05405		
58	20.79	C_33_H_40_O_15_	[M-H]^-^	675.22944	675.23138	4.951	Baohuoside VII	367.19135	352.19095	Flavonoids	H
								352.15344			
								323.26685			
59	21.02	C_32_H_38_O_14_	[M-H]^-^	645.21887	645.22021	3.763	Sagittatoside B	367.17920	352.13376	Flavonoids	H,S
								352.20306			
								323.13983			
60	21.09	C_33_H_40_O_14_	[M + H]^+^	661.24908	661.24774	−2.030	2″-O-rhamnosylikariside ΙΙ	515.16827	479.22516	Flavonoids	H
								497.22614	497.21771		
								479.21265	411.17444		
								369.21820			
61	21.48	C_27_H_30_O_10_	[M-H]^-^	513.17662	513.17822	5.255	Baohuoside Ι	366.19739	351.09555	Flavonoids	H,S
								351.24054	323.09329		
								323.09595	311.04443		
62	21.50	C_21_H_20_O_6_	[M + H]^+^	369.13326	369.13229	−2.045	Icaritin	313.11514	243.03632	Flavonoids	H,S
								243.16803	298.03275		
								135.00369	187.04146		
63	21.83	C_19_H_22_O_3_	[M-H]^-^	297.14961	297.15009	5.280	Neocryptotanshinone ΙΙ	182.91351	/	Phenanthraquinone	H,S
								277.04773			
								269.11948			
								297.16641			
64	22.02	C_19_H_16_O_4_	[M + H]^+^	309.11213	309.11154	−1.927	Tanshinone ΙΙB	265.12256	/	Diterpenoids	H,S
								291.12027			
								223.00299			
65	22.02	C_45_H_70_O_19_	[M-H]^-^	913.44385	913.44672	4.340	Pratioside D1 or its isomers	867.35022	/	Steroidal saponins	H
								807.52045			
								765.70355			
								729.80646			
66	22.31	C_19_H_22_O_4_	[M-H]^-^	313.14453	313.14536	6.145	Tanshinone V	269.13837	213.00495	Phenanthraquinone	H,S
								213.08423	226.08397		
								226.13559	241.19481		
								241.13177			
67	22.62	C_15_H_10_O_5_	[M-H]^-^	269.04554	269.04633	6.988	Emodin	225.01404	181.04298	Anthraquinones	H,S
								241.11241	210.09750		
								269.04004	225.15312		
								210.04666	197.19930		
								197.03294			
68	22.65	C_18_H_14_O_3_	[M + H]^+^	279.10157	279.10110	−1.687	Methylene tanshiquinone/DihydrotanshinoneⅠ	261.05334	233.07753	Diterpenoids	H,S
								233.07175	205.09665		
								251.14920	215.08347		
69	23.77	C_19_H_20_O_3_	[M + H]^+^	297.14852	297.14786	−2.225	Cryptotanshinone	279.13593	251.12753	Diterpenoids	H,S
								251.12753	237.13004		
									264.10468		
70	24.77	C_19_H_18_O_3_	[M + H]^+^	295.13287	295.13235	−1.765	Tanshinone ΙΙA	277.09225	/	Diterpenoids	H,S
								249.11270			
								266.03036			
								280.13669			
71	27.16	C_18_H_34_O_2_	[M-H]^-^	281.24860	281.24960	7.443	Oleic acid	261.12915	/	Others	H
								263.29987			
								237.12189			

MS2, secondary mass spectrometry fragment ions; MS3, tertiary mass spectrometry fragment ions; H, HLS; S, drug-containing serum.

Flavonoids have been identified in Yin Yanghuo and *Astragalus mongholicus* Bunge, and accordingly, a large number of flavonoids were identified in the HLS preparation, including flavonoid aglycones and flavonoid glycosides containing sugar groups. Flavonoid glycosides are prone to shedding of sugar groups; shedding of water molecules; reverse Diels-Alder reaction; and loss of CO, CO_2_, CHO, and other neutral molecules during the cleavage process (Cui et al., 2020). Molecule No.60, for example, had a quasi-molecular ion with *m/z* 661.24774 [M + H]^+^, and the molecular formula was determined to be C_33_H_40_O_14_. Its secondary fragment ion *m/z* 515.16827 was [M + H-C_6_H_10_O_4_]^+^, and *m/z* 369.21820 indicated [M + H-C_6_H_10_O_4_-C_6_H_10_O_4_]^+^. Combined with the database and the existing literature (Zhao et al., 2008), No.60 was identified as 2″-O-rhamnosylikariside II. The fragmentation diagram is shown in Supplementary Figure S1A. Phenolic acid compounds have been identified in *Salvia miltiorrhiza* Bunge and often protocatechuic aldehyde, danshensu, caffeic acid, and its dimer or polymer structures. Under the condition of the negative ion mode, danshensu (*m/z* 198), caffeic acid (*m/z* 179), and caffeoyl groups are often lost (Chen et al., 2020). A further example is No.38; the quasi-molecular ion had *m/z* 491.10071 [M-H]^-^, and the molecular formula was determined to be C_26_H_20_O_10_. Its secondary fragment ions were detected at *m/z* 311.06104 as [M-H-C_9_H_9_O_4_]^-^, *m/z* 293.11026 as [M-H-C_9_H_10_O_5_]^-^, *m/z* 265.04987 as [M-H-C_9_H_9_O_4_-COOH]^-^, and the tertiary fragment ion *m/z* 249.08069 as [M-H-C_9_H_10_O_5_-COOH]^-^. Combined with the database and literature (Li et al., 2008), No.38 was identified as salvianolic acid C. The fragmentation diagram is shown in Supplementary Figure S1D.

#### Bioactive Chemical Components Identified in Mouse Serum

The TIC diagram of the serum samples of the mice in the HLC treatment group is shown in Figure 2B (a 4 times enriched serum sample) and Figure 2C (a 2 times enriched serum sample). The TIC diagram of the serum samples of the mice in the blank group is shown in Figure 2D (a 4-fold enriched serum sample) and Figure 2E (a 2 times enriched serum sample). We combined the TIC maps of the blank mouse serum to exclude endogenous components and identified 31 migration components in the blood. The details are reported in Table 3 and Supplementary Date Sheet S1.

Considering No.67 as an example, the quasi-molecular ion had an *m/z* 269.04633 [M-H]^-^ value, and the molecular formula was identified as C_15_H_10_O_5_. Its secondary fragment ion *m/z* 241.11241 was determined to be [M-H-CO]^-^, and similarly, *m/z* 197.03294 was [M-H-CO-CO_2_]^-^, *m/z* 225.01404 was [M-H-CO_2_]^-^, and *m/z* 210.04666 was [M-H-CO_2_-CH_3_]^-^. Compared with the control substance and combined with the previous literature (Huang et al., 2018), No.67 was identified as emodin. The fragmentation diagram is shown in Supplementary Figure S1B.

For No.69, the quasi-molecular ion was *m/z* 297.14786 [M + H]^+^, and the molecular formula was determined to be C_19_H_20_O_3_. Its secondary fragment ion *m/z* 279.13593 was [M + H-H_2_O]^+^, and the tertiary fragment ion *m/z* 251.12753 was [M + H-H_2_O-CO]^+^. According to the characteristics of the fragment ion peaks and the literature report (Liang et al., 2017), No.69 was identified as cryptotanshinone. The fragmentation diagram is shown in Supplementary Figure S1E.

### Network Pharmacology and Molecular Docking of the Antioxidant Properties of HLS

#### Constructed Database of HLS Inflow Components

Through analysis and comparison, we preliminarily determined that the blood component library included 31 chemical components of HLS identified in mouse serum.

#### Component Targets

A total of 372 targets were obtained from 20 chemical components in the TCMSP database. We imported the SDF files of 10 chemical components obtained in the PubChem database for Swiss Target Prediction to obtain a total of 1,000 targets. After removing 1,372 repeated targets, this left 500 potential targets, which were transformed into 500 standard gene targets by the Uniprot database (Supplementary Date Sheet S2).

#### Disease Targets

In GeneCards, we obtained 134 genes with a score >20 using the two search terms related to antioxidants. For details, see Supplementary Date Sheet S3.

#### Common Targets

**FIGURE 4:**
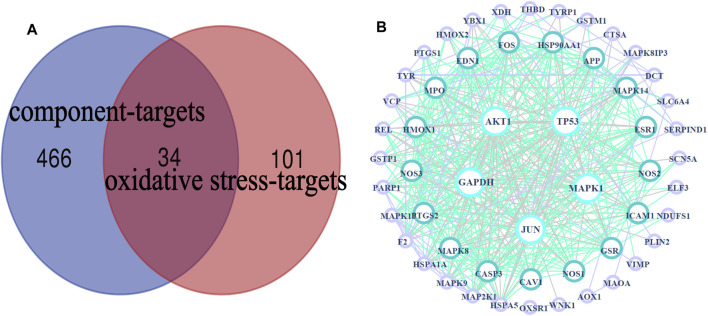
HLS target network. **(A)** Venn diagram of 34 potential “common targets” of HLS was intersected with the “component targets” and “oxidative stress targets,” **(B)** PPI network.

A total of 34 common targets were obtained after the intersection of component targets and disease targets. The Venn diagram and data table are shown in Figure 4A and Supplementary Date Sheet S4.

#### Indirect Targets

Under the condition of “*Homo sapiens*”, the common targets indicative of the antioxidant activity of HLS were introduced into GeneMANIA to obtain 20 indirect targets (Supplementary Date Sheet S4).

#### PPI Network Analysis

The PPI network obtained by uploading 54 targets to the String database was imported into Cytoscape 3.7.2 software for analysis, and visual images were obtained (Figure 4B), which represented the PPI relationship and contained 54 nodes and 410 edges. We used the “Dgree” value to perform topological analysis and screened the top five main targets: GAPDH, AKT1, TP53, MAPK1, and JUN.

#### Biological Function and Pathway Enrichment

**FIGURE 5:**
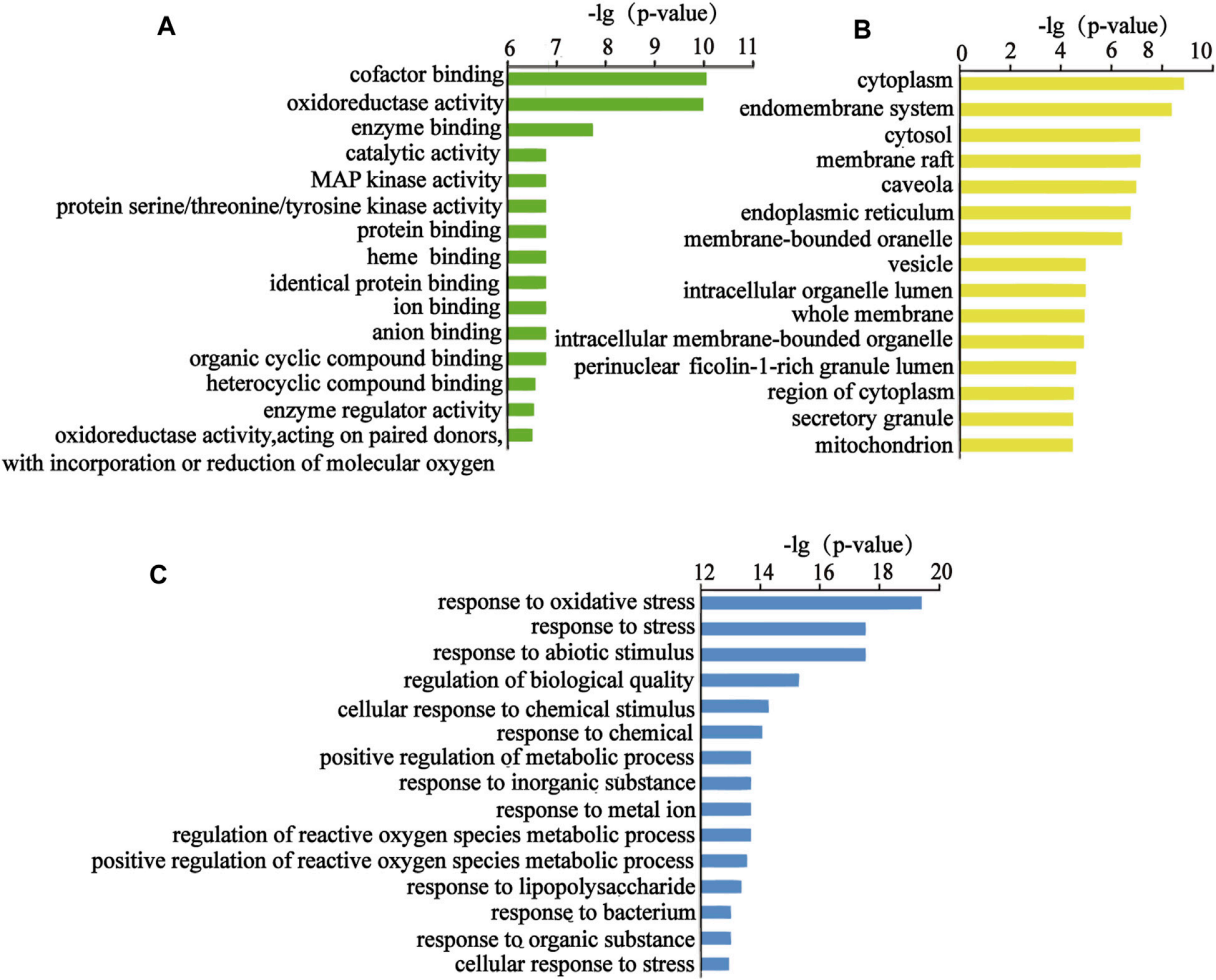
Top 15 items of the GO analysis. **(A)** Top 15 molecular functions, **(B)** top 15 cellular components, and **(C)** top 15 biological processes.

**FIGURE 6:**
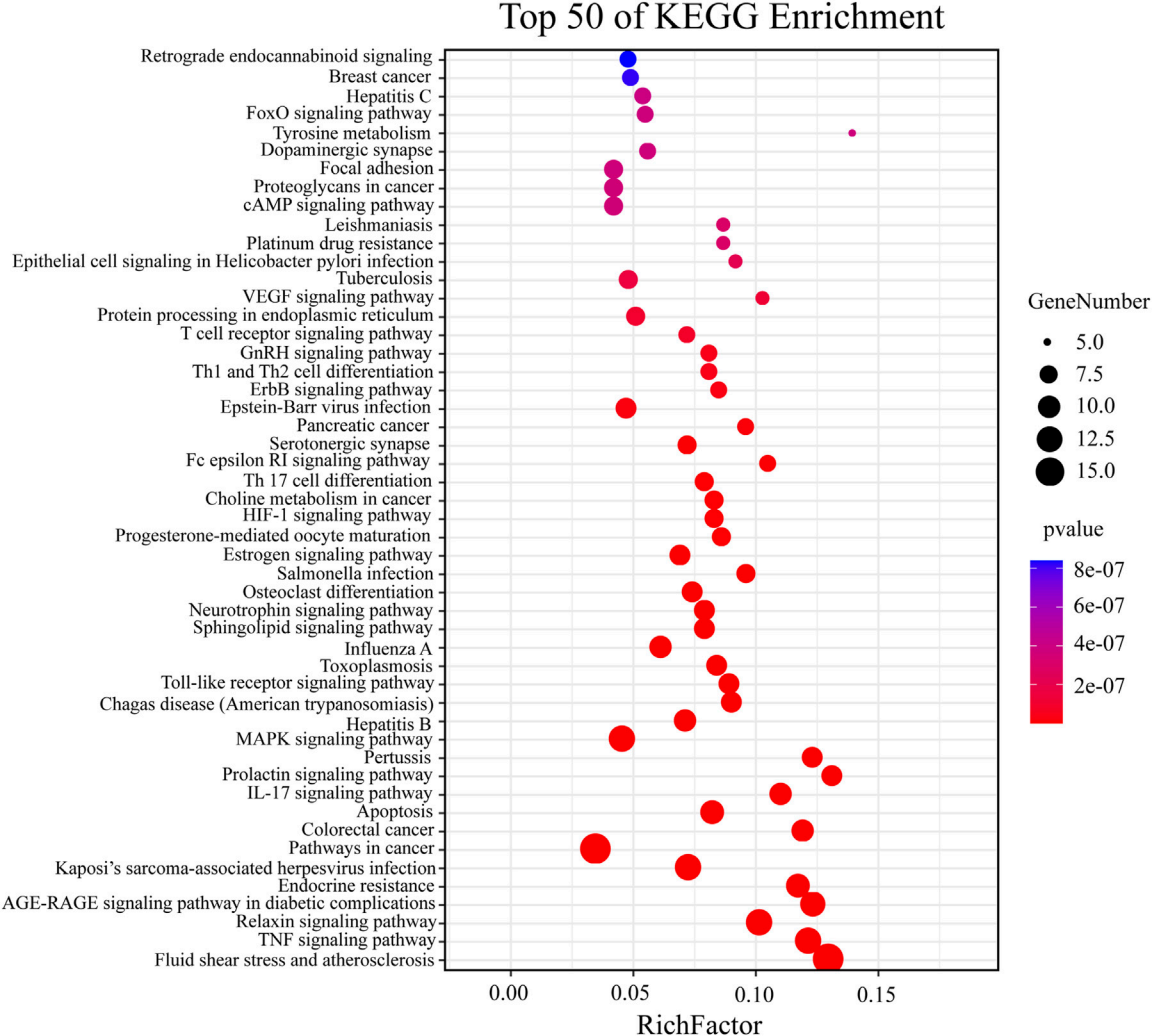
Enrichment analysis of KEGG pathways.

The above targets were imported into the String database, and the TSV format file describing the results of the target enrichment analysis was downloaded. A total of 962 biological processes, 59 cellular components, 103 molecular functions, and 145 Kyoto Encyclopedia of Genes and Genomes (KEGG) pathways were identified. Among these, the molecular function included “cofactor binding,” “oxidoreductase activity,” and “enzyme binding.” The biological process included “response to oxidative stress,” “response to stress,” and “response to abiotic stimulus.” “Cytoplasm,” “endomembrane system,” and “cytosol” were the cellular components identified as important components of cells. KEGG enrichment analysis was carried out using the String database, and the first 50 signal transduction pathways were visually analyzed by arranging the 145 KEGG pathways according to the *p*-value from the largest to the smallest. The KEGG pathways mainly involved the AGE-RAGE, MAPK, and interleukin (IL)-17 signaling pathways. The diagram illustrating the Gene Ontology (GO) analysis and the bubble diagram of KEGG enrichment analysis are shown in Figures 5, 6.

#### Components–Targets–Pathways Interactive Network

**FIGURE 7:**
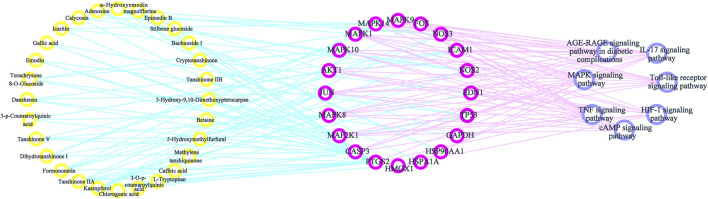
Components–targets–pathways network diagram.

By consulting the literature, seven important pathways related to antioxidative effects were identified. To directly show the relationship between the active components, targets, and KEGG pathways, we used Cytoscape 3.7.2 software to construct a network model of the active components–targets–pathways (Figure 7). The same component corresponded to multiple action targets, while multiple action targets corresponded to the same pathway.

#### Molecular Docking of Target Proteins With the Respective Reverse-Screened Chemical Components

**FIGURE 8:**
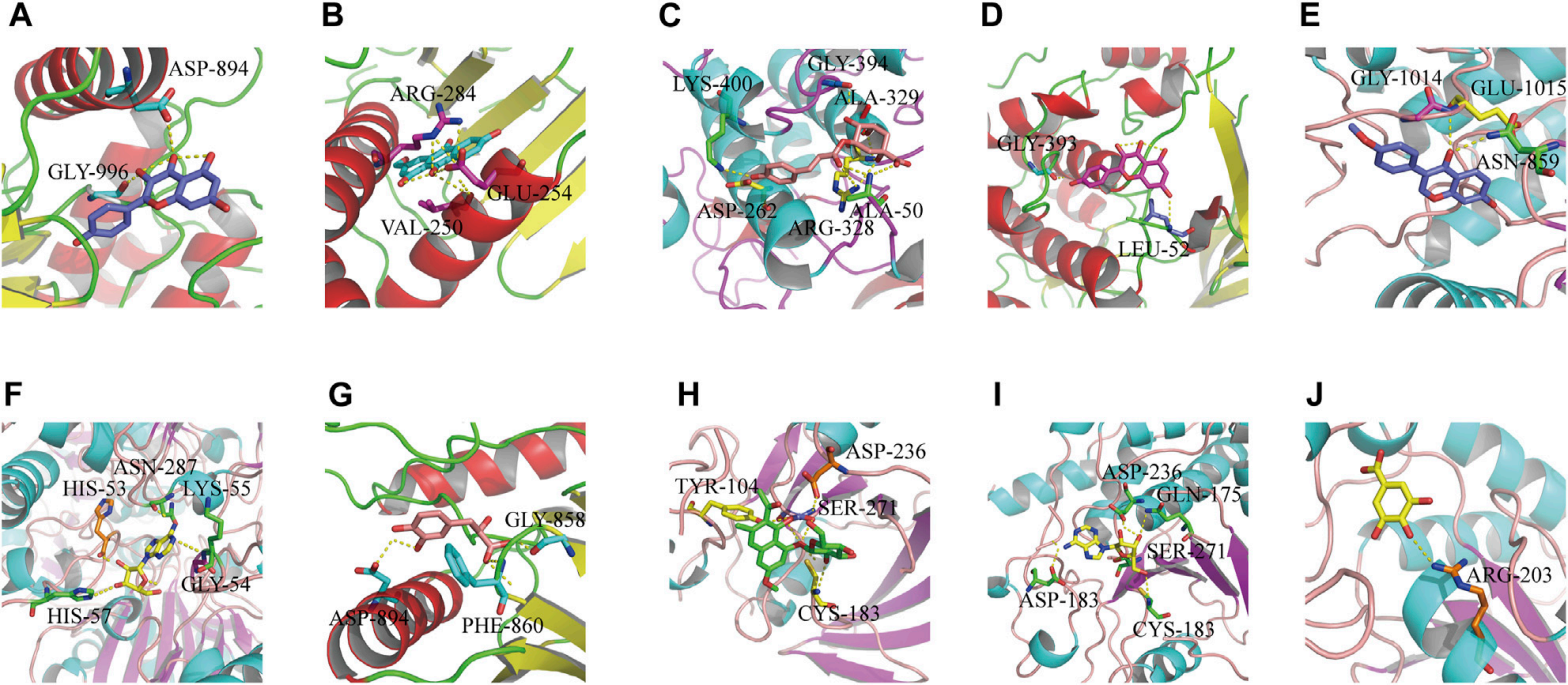
Results of molecular docking between target proteins and their reverse-screened chemical components. Optimal molecular docking of **(A)** kaempferol with JUN, **(B)** ω-hydroxyemodin with MAPK1, **(C)** 5-p-coumaroylquinic acid with AKT1, **(D)** kaempferol with AKT1, **(E)** formononetin with JUN, **(F)** adenosine with GAPDH, **(G)** danshensu with JUN, **(H)** torachrysone 8-O-glucoside with MAPK1, **(I)** adenosine with MAPK1, and **(J)** gallic acid with TP53.

We used the “Dgree” value to screen the top five main targets (GAPDH, AKT1, TP53, MAPK1, and JUN) for molecular docking with the corresponding components (kaempferol, formononetin, tanshinone ΙΙA, gallic acid, torachrysone-8-O-glucoside, 5-p-coumaroylquinic acid, adenosine, danshensu, and ω-hydroxyemodin). The results showed that the nine components had strong binding affinity with their corresponding targets (binding energy ≤ −7 kcal mol^−1^) (Supplementary Date Sheet S5). Molecular docking has preliminarily verified the core targets of HLS antioxidant effects. The results were visualized with PyMOL software and are shown in Figure 8 **.**

### Antioxidative Effects of HLS *in vivo*

**FIGURE 9:**
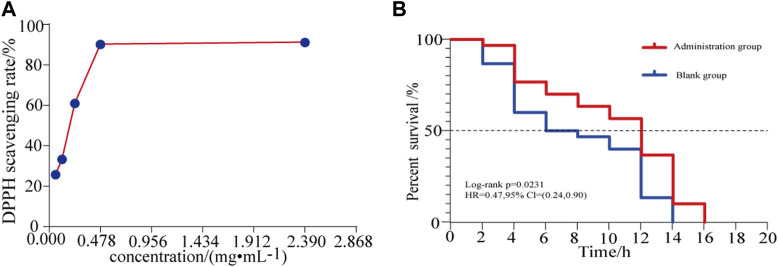
**(A)** Effects of different concentrations of HLS on the clearance rate of DPPH and **(B)** antioxidant survival curve of *C. elegans* under the activity of juglone.

The DPPH free radical scavenging ability of different concentrations of HLS was determined, and the results are summarized in Figure 9A and Supplementary Date Sheet S6. The DPPH radical scavenging rate increased with the increasing concentration of the test solution in a dose-dependent manner. When the sample solution reached a certain concentration, the clearance rate increased slowly. When the HLS test sample was diluted 10 times, the clearance rate reached 91.29%.

### Antioxidative Effects of HLS *in vitro*

In a model of oxidative damage induced by juglone, the log-rank test revealed that the survival curve of *C. elegans* under oxidative stress was significantly different (*p* = 0.00231). As shown in Figure 9B, for each measurement, the survival of the treatment group was longer than that of the controls, and the ability to resist oxidative stress generally improved. This confirmed that HLS could enhance the ability of *C. elegans* to resist oxidative stress.

## Discussion

### Antioxidative Effects of HLS

Aging refers to the rational aging of the human body over time and is accompanied by pathological changes, such as cardiovascular diseases, neurodegenerative diseases, diabetes, cancer, and other cognitive diseases (López-Otín et al., 2013; Carmona and Michan, 2016; Liang et al., 2017). Delaying the aging of the body and improving the quality of life of the elderly have become important research objectives worldwide (Zhang et al., 2020). According to the free radical theory, when the body’s ability to scavenge free radicals weakens, it leads to the accumulation of reactive oxygen species (ROS) and oxidative damage to the body, which in turn leads to protein and nucleic acid denaturation and lipid peroxidation, and finally leads to aging (Navarro-Yepes et al., 2014; Sharma et al., 2018). Therefore, reducing the level of free radicals in the body with therapeutic agents having antioxidant activity has become an effective way to delay aging. DPPH is a very stable free radical centered on the nitrogen atom. It has a characteristic absorption at the wavelength of 517 nm. Its alcohol solution is dark purple in color. After adding a test substance, the absorbance value changes due to the reduction of the DPPH free radical. The free radical scavenging rate can be calculated by the change in ratio of absorbance values (Qiu et al., 2016). This method is simple, sensitive, and reproducible and is widely used for the detection of antioxidants (Zhang et al., 2010; Zhang et al., 2016). Thus, the DPPH free radical scavenging test was used in this study to evaluate the antioxidant activity of HLS *in vitro*. Our findings indicated that when the concentration of HLS was 0.059 mg/mL^−1^ to 0.478 mg/mL^−1^, the free radical scavenging rate increased from 25.84 to 90.31%. When the concentration was 2.39 mg/mL^−1^, the scavenging rate reached 91.29%. Thus, HLS exerted antioxidative activity *in vitro*, and it was positively correlated with the concentration. The antioxidant capacity of *C. elegans* was closely related to its survival time, which can be used as an index to screen whether the drug exerted any antiaging effects (Kaletta and Hengartner, 2006; Zhou et al., 2018). When *C. elegans* is exposed to juglone, it produces a large amount of oxygen free radicals in a short period of time, causing acute oxidative damage and ultimately leading to the death of the *C. elegans* (Link and Wink, 2019). In this study, an oxidative stress condition of 400 μmol L^−1^ juglone was used to increase the active oxygen levels in *C. elegans*, and a *C. elegans* oxidative stress model was obtained. By comparing the survival times of *C. elegans* in the control group and the treatment group, the survival curve of *C. elegans* in the treatment group was shifted significantly to the right, indicating that HLS significantly increased the survival time of *C. elegans* under the oxidative stress mode, increased antioxidant capacity, and prolonged the lifespan. It has been reported that polyphenols contained in *Salvia miltiorrhiza* Bunge in HLS can improve the antioxidant ability of cells and inhibit the production of ROS (Fei et al., 2013). Flavonoids in Yin Yanghuo can scavenge free radicals, increase the level of antioxidant enzymes, and reduce the production of ROS (Li et al., 2018). Therefore, we speculated that HLS may achieve antiaging activity by scavenging active oxygen free radicals in the body, by improving antioxidant enzyme activity, and by enhancing antioxidant capacity. In this study, the antioxidant activity of HLS was comprehensively evaluated through its *in vivo* and *in vitro* antioxidant activity, which proved that HLS had good ability of scavenging free radicals and exerting antiaging effects.

### Antioxidant Components of HLS Screened Based on Serum Pharmacochemistry and Network Pharmacology

The serum pharmacochemistry of TCM uses the UPLC-LTQ-Orbitrap MS technology to analyze the components of TCM that enter the blood circulation after oral administration. Network pharmacology can be used to predict and identify the effective component groups of TCM to treat diseases (Song et al., 2014) and to determine whether the components entering the body are effective agents. In recent years, most of the network pharmacology studies reported in the literature (Hung et al., 2021; Jiang et al., 2021; Zhang et al., 2021) were based on the oral bioavailability of the ingredients listed in their databases as the screening index. However, given the complex formulation of different TCM preparations, the bioavailability of some components may change under the mutual influence of other components, while changes in the technology used to prepare TCM will also change the bioavailability of different components (Li et al., 2016). Thus, for compound prescriptions of TCM that comprise multicomponents, using oral bioavailability as a screening index will lead to insufficient target prediction. Using serum medicinal chemistry to determine the active components of HLS that directly act in the body will achieve a more accurate target prediction than simply relying on oral bioavailability data from a screening database as an indicator (Wang et al., 2020). This study established a research model combining serum medicinal chemistry and network pharmacology and proposed new ideas for further research into elucidating the active groups of TCM compounds.

In this study, the antioxidant active components of HLS were screened based on serum medicinal chemistry and network pharmacology techniques. First, the UPLC-LTQ-Orbitrap MS technology was used to analyze and compare the methanol extract of HLS, a blank mouse serum, and the mouse serum obtained after intragastric administration of HLS. In total, 71 chemical components in the HLS and 31 prototype components in serum samples were determined. Second, based on network pharmacology, 29 active antioxidant components of HLS were reverse-screened based on the composition–disease target network diagram. Based on the chemical composition, these were divided into flavonoids, diterpenoids, phenolic acids, quinones, and alkaloids, and of these, flavonoids comprise the largest group, followed by phenolic acids and diterpenoids. Studies have shown that kaempferol, a flavonoid component, increases the activity of antioxidant enzymes in the serum of model rats, reduces the content of lipid peroxidation intermediates, and has central antioxidant effects (Zhang et al., 2019). Gallic acid, a component of phenolic acids, resists oxidative stress in the nervous system (Qin et al., 2020). Tanshinone ІІA, a diterpenoid component, can reduce ROS and malondialdehyde levels in the serum of patients with type ІІ diabetes and increases the antioxidant indexes glutathione and superoxide dismutase to exert an antioxidant role (Mei and Zhang, 2014). Emodin, a quinone component, antagonizes renal oxidative damage caused by hypertension by increasing superoxide dismutase activity and reducing malondialdehyde content (Ma et al., 2018). Altogether, considering the literature and the blood ingredients–disease targets network map, we speculate that HLS exerts antioxidant activity, which is accomplished by multiple antioxidant components jointly regulating multiple targets and related pathways.

### Molecular Mechanisms of Antioxidant Effects of HLS

Inflammation is closely associated with oxidative stress. Oxidative stress is caused by an increase in ROS production and by a decrease in the ability of scavenging ROS. ROS can promote the production of AGEs, which mediate a variety of signaling pathways triggered by cell membrane surface receptors, including RAGE. The AGEs-RAGE signaling pathway causes nicotinamide adenine dinucleotide phosphate (NADPH) oxidase to be activated to produce a large amount of ROS, which in turn activates the MAPK signaling pathway implicated in cell survival, proliferation, differentiation, and apoptosis. This process activates the oxidative stress-sensitive transcription factor NF-κB to release a variety of chemokines, inflammatory factors, and adhesion molecules, such as MCP-1, PAI-1, ICAM-1, IL-1, IL-6, and IL-8 (Xu et al., 2012). Furthermore, the activation of the transcription factor NF-κB by oxidative stress leads to increased IL-17 expression and thus a feedback loop that further activates the NF-κB signaling pathway (Spadari et al., 2018). IL-17 is a cellular inflammatory factor that induces inflammatory responses *in vivo*. It can promote the expression of downstream cytokines IL-6 and tumor necrosis factor (TNF)-α. As a pathogenic factor, TNF-α activates the downstream MAPK signaling pathway and NF-κB signaling pathway, releases pro-inflammatory factors, and exerts a pro-inflammatory activity via the TNF signaling (Micheau and Tschopp, 2003). Toll-like receptors (TLRs) are a class of important protein molecules involved in non-specific immunity. The activation of the NF-κB pathway produces a variety of inflammatory cytokines, which do not only directly damage cells but also lead to the amplification and persistence of inflammatory cascades. The persistent inflammatory response induces oxidative stress in the body to further cause damage. In summary, HLS may exert antioxidant activity by controlling the release of inflammatory cytokines: the active ingredients act on the AGE-RAGE signaling pathway through MAPK1, MAPK14, ICAM1, NOS3, and CASP3 and reduce the production of ROS. Through MAPK1, MAPK14, TP53, MAP2K1, and FOS, the MAPK signaling pathway controls the release of inflammatory factors. The active ingredients activate MAPK1, MAPK14, FOS, CASP3, and HSP90AA1, which stimulate the IL-17 signaling pathway, and control the release of inflammatory factors. In addition, active ingredients activate MAPK1, MAPK14, ICAM1, MAP2K1, and FOS and activate the TNF signaling pathway to control the release of inflammatory factors. Finally, active ingredients also activate the TLR signaling pathway through MAPK1, MAPK14, MAP2K1, FOS, and MAPK10 to stimulate TLR signaling and the regulation of the inflammatory response and the release of inflammatory factors.

Mitochondria can consume ROS and exert antioxidant activity (Lim and Luderer, 2011). When excessive ROS is produced in the body, this leads to lipid peroxidation and structural destruction of the mitochondrial cell membrane. Cells can selectively remove damaged or excess mitochondria through autophagy. Autophagy is closely associated with the accumulation of mitochondrial oxidative damage (Ikeda et al., 2014). The cyclic adenosine monophosphate (cAMP) signaling pathway regulates the metabolism and mitochondrial oxidation of lipids. HLS may act on the cAMP signaling pathway through targets such as MAPK1, MAP2K1, FOS, MAPK10, JUN, MAPK8, MAPK9, and AKT1. Hypoxia-inducible factor (HIF)-1α is an active subunit of HIF-1 present in mitochondria. It not only inhibits the production of ROS but also promotes mitochondrial autophagy through the BNIP3/Bcl-2 pathway. HLS, via MAPK1, HMOX1, GAPDH, NOS3, MAP2K1, and other targets, may act on the HIF-1 signaling pathway to enhance autophagy and exert antioxidant effects.

Altogether, the occurrence of oxidative stress may be related to inflammatory reactions, the release of inflammatory cytokines, cell mitophagy, and metabolic abnormalities. The molecular mechanisms of the antioxidant effects of HLS involve the regulation of multiple targets and multiple pathways.

### Experimental Evaluation

In this study, the animal models received high doses of HLS, which were much greater than the clinically equivalent doses in order to achieve stable blood–drug concentrations and to ensure that the drug components in the serum were present at higher levels, thus allowing them to be easily identified. The specific time point for blood collection in mice was set at 30 and 60 min after administration to make the results more accurate and comprehensive. However, due to the complexity of the components of TCM, some components may not have been absorbed or may have been excreted during this interval, and thus, they may not have been detected. However, there are some shortcomings that need to be examined further. With regard to the research technology, although network pharmacology is based on the analysis of big data and systems biology to integrate the information of multiple compounds, network pharmacology is still limited by available biological information on the platform and recorded in the database and likely does not comprehensively contain all relevant information. In addition, network pharmacology ignores the dose–effect relationship of multiple components in TCM compounds. We will improve these shortcomings in future experiments. With regard to experimental verification, we have conducted preliminary verification of the targets and components of network pharmacology screening through molecular docking, but we still need relevant biological experimental data as support. In the future, we plan to carry out relevant cell and animal experiments to further verify the key ways of antioxidants, with a view to developing important compounds into medicinal substances or applying extracts to treatments. The DPPH experiment proved the antioxidant effect of HLS from a chemical point of view. In the future, we will determine the IC50 value to further prove the antioxidant effect of HLS from the pharmacological aspect.

## Conclusions

In this study, we confirmed the antioxidant activity of HLS using *C. elegans* as a model organism and DPPH studies. Based on serum pharmacochemistry and network pharmacology, we systematically explored the basis of the antioxidant activity of HLS and discussed the potential molecular mechanisms involved. We found that HLS regulates GAPDH, AKT1, TP53, MAPK1, JUN, and other related targets and influences the TAGE-RAGE, MAPK, and IL-17 signaling pathways, thereby reducing inflammation and controlling the release of inflammatory cytokines and regulating mitophagy and metabolic abnormalities, all of which ultimately play an antioxidant role. This study fully embodies the synergistic characteristics of multiple components, multiple targets, and multiple pathways of TCM compounds and lays a foundation for exploring the antioxidant mechanism of HLS. Further, our findings provide a theoretical basis for the future application of HLS in clinical antiaging prevention strategies.

## Data Availability

The raw data supporting the conclusions of this article will be made available by the authors, without undue reservation, to any qualified researcher.

## Abbreviations

*C. elegans*, *Caenorhabditis elegans*; *E. coli* OP50, *Escherichia coli* OP50; GO, Gene Ontology; HESI, heated electrospray ionization; HLS, Huolisu Oral Liquid; KEGG, Kyoto Encyclopedia of Genes and Genomes; PDB, Protein Data Bank; DPPH, 1,1-dipheny-2-picrylhydrazyl; PPI, protein–protein interaction; TCM, traditional Chinese medicine; TCMSP, Traditional Chinese Medicine Systems Pharmacology; TIC, total ion chromatograms; ROS, reactive oxygen species; UPLC-LTQ-Orbitrap MS, ultra-high-performance liquid chromatography coupled with electrospray ionization hybrid linear trap quadrupole orbitrap high-resolution mass spectrometry; Yin Yanghuo, *Epimedium brevicornu* Maxim.
