# Supplementary material for: The Molecular Mechanism of Antioxidation of Huolisu Oral Liquid Based on Serum Analysis and Network Analysis
Source: Front Pharmacol. 2021 Nov 1;12:710976. doi: 10.3389/fphar.2021.710976 (PMC8591200; doi:10.3389/fphar.2021.710976)
Supplement: Supplementary file 1 [file DataSheet1.zip › Schedule.doc]

***Supplementary Material***

**Contents:**

Supplementary Material 1 Huolisu Oral Liquid’s procedure

Supplementary Date Sheet S1 Dentification of the bioactive chemical prototype components in serum by LTQ-orbitrap-MS

Supplementary Date Sheet S2 The results of converting targets into official gene symbols

Supplementary Date Sheet S3 The Results of anti-oxidative targets on online database

Supplementary Date Sheet S4 Potential direct targets and indirect targets of anti-oxidant induced by HLS

Supplementary Date Sheet S5 Molecular docking of target proteins and its reverse selected chemical components

Supplementary Date Sheet S6 Results of DPPH free radical scavenging experiment

Supplementary Figure S1 The mass fragment and fragmentation pathway of some compounds

**Supplementary Material 1.** Huolisu Oral Liquid’s procedure

Huolisu Oral Liquid is a Chinese patent medicine approved and supervised by CFDA under the same strict quality control standards. Meanwhile, Huolisu Oral Liquid in our study have come from the same preparation. The details are as follows (Committee 2020):

**Ingredients:** *Reynoutria multiflora* (Thunb.) Moldenke [Polygonaceae; Polygoni multiflora radix praeparata], *Epimedium brevicornu* Maxim. [Berberidaceae; Epimedii folium], *Polygonatum sibiricum* F. Delaroche	[Asparagaceae; Polygonati rhizoma], *Lycium chinense* Mill. [Solanaceae; Lycii fructus], *Astragalus mongholicus* Bunge [Leguminosae; Astragali radix]*,* and *Salvia miltiorrhiza* Bunge [Lamiaceae; Salviae miltiorrhizae radix et rhizoma]

**Procedure:** Decoct polygoni multiflora radix praeparata, salviae miltiorrhizae radix et rhizoma, lycii fructus with water three times, 2 hours for the first time, 1.5 hours for the second and third time, respectively, filter, combine the filtrate, concentrate the filtrate to a thin extract with a relative density of 1.20-1.25 (60°C), allow to cool. Add ethanol to a content of 70%, allow to stand and filter. Add ethanol to a content of 80%, allow to stand and filter. add 10% solution of soldium hydroxide to adjust to pH 8.0, allow to stand and filter. Add 10% solution of hyrdrochloric acid to adjust to pH 7.0, recover ethanel to obtain the reserved extract for later use. Decoct epimedii folium and polygonati rhizoma with water twice, 2 hours for the first time, 1 hour for the second, filter, combine the filtrate, concentrate the filtrate to a thin extract with a relative density of 1.18-1.20 (60°C), cool, add ethanol to content of 65%, allow to stand and filter. Add ethanol to a content of 80%, allow to stand and filter. Add 10% solution of soldium hydroxide to adjust to pH 8.0, allow to stand and filter. Add 10% solution of hydrochloric acid to adjust to pH 7.0, recover ethanol to obtain the reserved extract for later use. Detect astragali radix with water three times, 2 hours for the first time, 1.5 hours for the second and third time respectively, filter, combine the filtrate and contentrate to 95 mL to obtain the reserved extract for later use. Combine all above reserved extract, stir well, allow to cool, filter, add water to 1000 mL, filter, add 10% solution of soldium hydroxide to adjust to pH 7.0-7.5.

**Supplementary Date Sheet S1.** Dentification of the bioactive chemical prototype components in serum by LTQ-orbitrap-MS

| **No** | **Rt (min)** | **Ions** | **Calcd**  ***m/z*** | **Obsed**  ***m/z*** | **Error (ppm)** | **Formula** | **MS^2^** | **Identify** | **Pubchem CID** | **From** | **Structure** |
| --- | --- | --- | --- | --- | --- | --- | --- | --- | --- | --- | --- |
| 1 | 1.02 | [M+H]^+^ | 118.08625 | 118.08651 | 2.158 | C_5_H_11_NO_2_ | 118.06535,  72.08051 | Betaine | 247 | B1 |  |
|  | 1.03 |  |  | 118.08658 | 2.751 |  | 118.08649,  72.08144 |  |  | C1 |  |
| 2 | 1.09 | [M+H]^+^ | 268.10403 | 268.10394 | -0.337 | C_10_H_13_N_5_O_4_ | 136.06175 | Adenosine | 60961 | B1 |  |
|  | 1.09 |  |  | 268.10406 | 0.110 |  | 136.06181 |  |  | C1 |  |
| 3 | 3.45 | [M+H]^+^ | 127.03897 | 127.03922 | 1.963 | C_6_H_6_O_3_ | 126.95541,  128.04236 | 5-Hydroxymethylfurfural | 237332 | B1 |  |
|  | 3.45 |  |  | 127.03925 | 2.199 |  | 127.03917,  128.04282 |  |  | C1 |  |
| 4 | 3.68 | [M-H]^-^ | 169.01424 | 169.01341 | 1.540 | C_7_H_6_O_5_ | 125.02319,  151.00261,  107.01251,  97.02821 | Gallic acid | 370 | B2 |  |
|  | 3.68 |  |  | 169.01343 | 0.280 |  | 125.02322,  151.00281,  107.01254,  97.95905 |  |  | C2 |  |
| 5 | 3.79 | [M+H]^+^ | 205.09715 | 205.09712 | -0.167 | C_11_H_12_N_2_O_2_ | 188.07053,  146.05997,  144.08076,  142.06509 | L-tryptophan | 6305 | B1 |  |
|  | 3.83 |  |  | 205.09717 | 0.077 |  | 188.07060,  146.06004,  144.08084,  142.06509 |  |  | C1 |  |
| 6 | 4.76 | [M-H]^-^ | 179.03498 | 179.03401 | 0.125 | C_9_H_8_O_4_ | 134.03635,  106.93391 | Caffeic acid | 689043 | B2 |  |
|  | 4.75 |  |  | 179.03409 | 1.144 |  | 134.98700,  106.93460 |  |  | C2 |  |
| 7 | 6.90 | [M-H]^-^ | 353.08780 | 353.08804 | 3.771 | C_16_H_18_O_9_ | 191.07042 | Chlorogenic acids | 1794427 | B2 |  |
|  | 6.67 |  |  | 353.08643 | -0.789 |  | 191.07057 |  |  | C2 |  |
| 8 | 6.82 | [M-H]^-^ | 337.09289 | 337.09283 | 3.074 | C_16_H_18_O_8_ | 173.04462,  163.03902 | 5-p-coumaroylquinic acid or 3-O-p-coumaroylquinic acid | 164893 or 9945785 | B2 |  |
|  | 6.82 |  |  | 337.09326 | 1.466 |  |  |  |  | C2 |  |
| 9 | 7.54 | [M+H]^+^ | 342.16998 | 342.17010 | 0.337 | C_20_H_23_NO_4_ | 297.11188,  265.08572,  311.12674,  282.08847 | Magnoflorine | 73337 | B1 |  |
|  | 7.54 |  |  | 342.17017 | 0.541 |  | 297.11191,  265.08569,  311.12729,  282.08844 |  |  | C1 |  |
| 10 | 9.30 | [M-H]^-^ | 593.15119 | 593.15112 | 1.024 | C_27_H_30_O_15_ | 300.00317 | Quercetin 3,7-dirhamnoside | 15953752 | B2 |  |
|  | 9.31 |  |  | 593.15143 | 1.334 |  |  |  |  | C2 |  |
| 11 | 9.35 | [M-H]^-^ | 405.11910 | 405.11887 | 2.126 | C_20_H_22_O_9_ | 137.02336,  173.06032,  225.05589,  243.06595 | Hyperoside | 5281643 | B2 |  |
|  | 9.36 |  |  | 405.11917 | 1.161 |  | 137.02336,  173.06032,  225.05589,  243.06596 |  |  | C2 |  |
| 12 | 10.95 | [M-H]^-^ | 197.04554 | 197.04477 | 1.625 | C_9_H_10_O_5_ | 179.07069,  153.09106,  135.08052,  123.00741 | DanShenSu | 11600642 | B2 |  |
|  | 10.98 |  |  | 197.04480 | 0.350 |  | 178.88335,  153.09108,  135.08035,  123.00755 |  |  | C2 |  |
| 13 | 11.54 | [M-H]^-^ | 493.11402 | 493.11563 | 2.707 | C_26_H_22_O_10_ | 295.26462 | Salvianolic acid | 5281793 | B2 |  |
|  | 11.76 |  |  | 493.11386 | 1.960 |  | 295.30099 |  |  | C2 |  |
| 14 | 12.12 | [M+H]^+^ | 301.10705 | 301.10715 | 0.332 | C_17_H_16_O_5_ | 286.08246,  176.04649 | 3-Hydroxy-9,10-Dimethoxypterocarpan | 5319733 | B1 |  |
|  | 12.13 |  |  | 301.10709 | 0.132 |  | 286.08228,  176.04678 |  |  | C1 |  |
| 15 | 12.33 | [M-H]^-^ | 283.06119 | 283.06125 | 4.063 | C_16_H_12_O_5_ | 268.03775,  239.03453,  225.05522 | Calycosin | 5280448 | B2 |  |
|  | 12.35 |  |  | 283.06125 | 1.150 |  | 268.03824,  239.03474,  225.05472 |  |  | C2 |  |
| 16 | 12.50 | [M+H]^+^ | 809.28625 | 809.28711 | 1.056 | C_38_H_48_O_19_ | 369.13333,  313.07022 | Epimedin B | 5748393 | B1 |  |
|  | 12.50 |  |  | 809.28693 | 0.833 |  | 369.35187,  313.07086 |  |  | C1 |  |
| 17 | 12.94 | [M-H]^-^ | 431.09837 | 431.09842 | 1.147 | C_21_H_20_O_10_ | 269.04553,  311.05695,  293.04541,  225.05540 | Torachrysone 8-O-glucoside | 11972479 | B2 |  |
|  | 12.96 |  |  | 431.09830 | 1.027 |  | 269.04550,  225.05516 |  |  | C2 |  |
| 18 | 13.03 | [M+H]^+^ | 677.24399 | 677.24384 | -0.232 | C_33_H_40_O_15_ | 369.13306,  313.07034 | Icariin | 5318997 | B1 |  |
|  | 13.04 |  |  | 677.24396 | -0.054 |  | 369.13300,  313.07025 |  |  | C1 |  |
| 19 | 15.77 | [M-H]^-^ | 285.04046 | 285.04050 | 3.984 | C_15_H_10_O_6_ | 241.05043,  285.04050 | ω- Citreorosein | 361512 | B2 |  |
|  | 15.79 |  |  | 285.04034 | 0.976 |  | 241.05028,  285.04025 |  |  | C2 |  |
| 20 | 15.77 | [M-H]^-^ | 285.04046 | 285.04050 | 3.984 | C_15_H_10_O_6_ | 241.05043,  285.04050 | Kaempferol | 5280863 | B2 |  |
|  | 15.79 |  |  | 285.04034 | 3.422 |  | 241.05028,  285.04025 |  |  | C2 |  |
| 21 | 19.11 | [M-H]^-^ | 267.06628 | 267.06610 | 3.425 | C_16_H_12_O_4_ | 222.89885,  267.06631,  225.05504 | Formononetin | 5280378 | B2 |  |
|  | 19.14 |  |  | 267.06604 | 3.200 |  | 267.06631,  225.05554 |  |  | C2 |  |
| 22 | 20.28 | [M-H]^-^ | 645.21887 | 645.21887 | 1.686 | C_32_H_38_O_14_ | 367.11774,  352.09702,  323.09338 | Sagittatoside B | 10146160 | B2 |  |
|  | 20.29 |  |  | 645.21887 | 1.686 |  |  |  |  | C2 |  |
| 23 | 20.75 | [M-H]^-^ | 513.17662 | 513.17621 | 1.338 | C_27_H_30_O_10_ | 366.11075,  351.08740,  323.09238,  311.05588 | Baohuoside Ι | 5488822 | B2 |  |
|  | 20.76 |  |  | 513.17657 | 2.039 |  | 366.11075,  351.08734,  323.09238,  311.05618 |  |  | C2 |  |
| 24 | 20.75 | [M+H]^+^ | 369.13326 | 369.13312 | -0.392 | C_21_H_20_O_6_ | 313.07047,  243.06506,  135.04413,  187.07530 | Icaritin | 5318980 | B1 |  |
|  | 20.75 |  |  | 369.13293 | -0.907 |  | 313.07028,  243.06500,  135.04414,  187.07518 |  |  | C1 |  |
| 25 | 21.23 | [M+H]^+^ | 279.10157 | 279.10141 | -0.161 | C_18_H_14_O_3_ | 261.22141,  233.09706 | Methylenetanshinquinone/Dihydrotanshinone Ι | [105118](https://pubchem.ncbi.nlm.nih.gov/compound/105118) or [11425923](https://pubchem.ncbi.nlm.nih.gov/compound/11425923) | B1 |  |
|  | 21.23 |  |  | 279.10126 | -1.114 |  | 261.22125,  233.87679 |  |  | C1 |  |
| 26 | 21.27 | [M+H]^+^ | 309.11213 | 309.11185 | -0.924 | C_19_H_16_O_4_ | 265.12216,  291.10120,  223.07533 | Tanshinone ΙΙB | [318797](https://pubchem.ncbi.nlm.nih.gov/compound/318797) | B1 |  |
|  | 21.27 |  |  | 309.11191 | -0.730 |  | 265.12216,  291.10135,  223.07532 |  |  | C1 |  |
| 27 | 21.56 | [M-H]^-^ | 313.14453 | 313.14417 | 2.345 | C_19_H_22_O_4_ | 213.12759,  269.15451,  226.09952,  241.15956 | Tanshinone V | [184103](https://pubchem.ncbi.nlm.nih.gov/compound/184103) | B2 |  |
|  | 21.56 |  |  | 313.14438 | 3.016 |  | 213.09178,  269.15445,  226.09952,  241.15968 |  |  | C2 |  |
| 28 | 21.57 | [M+H]^+^ | 297.14852 | 297.14825 | -0.912 | C_19_H_20_O_3_ | 251.14296,  237.09109,  264.11447 | Cryptotanshinone | [160254](https://pubchem.ncbi.nlm.nih.gov/compound/160254) | B1 |  |
|  | 21.56 |  |  | 297.14807 | -1.518 |  | 251.14262,  237.09087,  264.11313 |  |  | C1 |  |
| 29 | 21.87 | [M-H]^-^ | 269.04554 | 269.04532 | 3.234 | C_15_H_10_O_5_ | 269.04544,  225.05518,  241.05026,  210.03166,  197.05997,  181.06522 | Emodin | 3220 | B2 |  |
|  | 21.88 |  |  | 269.04538 | 3.457 |  | 269.21231,  225.18457,  181.15866 |  |  | C2 |  |
| 30 | 22.64 | [M-H]^-^ | 297.14961 | 297.14923 | 2.386 | C_19_H_22_O_3_ | 297.24332 | Neocryptotanshinone ΙΙ | [5320066](https://pubchem.ncbi.nlm.nih.gov/compound/5320066) | B2 |  |
|  | 22.64 |  |  | 297.14966 | 3.833 |  | 297.24280 |  |  | C2 |  |
| 31 | 23.98 | [M+H]^+^ | 295.13287 | 295.13303 | 0.539 | C_19_H_18_O_3_ | 277.12173,  231.17326 | Tanshinone ΙΙA | [14609844](https://pubchem.ncbi.nlm.nih.gov/compound/14609844) | B1 |  |
|  | 24.00 |  |  | 295.13263 | -0.816 |  | 277.21713,  231.21066 |  |  | C1 |  |

Note: B1: group enriched with 4-fold serum（HESI^+^）, B2: group enriched with 4-fold serum（HESI^-^）, C1: group enriched with 2-fold serum（HESI^+^）, C2: group enriched with 2-fold serum（HESI^-^）.

**Supplementary Date Sheet S2.** The results of converting targets into official gene symbols

| **No** | **Components** | **Target** | **Symbol** |
| --- | --- | --- | --- |
| 1 | 3-O-p-coumaroylquinic acid | 8 | 6 |
| 2 | 5-Hydroxymethylfurfural | 20 | 15 |
| 3 | epimedin B | 24 | 20 |
| 4 | L-tryptophan | 9 | 5 |
| 5 | methylenetanshinquinone | 24 | 19 |
| 6 | formononetin | 39 | 34 |
| 7 | Emodin | 35 | 30 |
| 8 | tanshinone ΙΙA | 41 | 37 |
| 9 | hyperoside | 9 | 7 |
| 10 | sagittatoside B | 10 | 7 |
| 11 | caffeic acid | 9 | 9 |
| 12 | Chlorogenic acid | 1 | 1 |
| 13 | calycosin | 22 | 20 |
| 14 | gallic acid | 15 | 13 |
| 15 | magnoflorine | 24 | 22 |
| 16 | anhydroicaritin | 23 | 20 |
| 17 | kaempferol | 63 | 58 |
| 18 | betaine | 32 | 21 |
| 19 | Icariin | 1 | 1 |
| 20 | cryptotanshinone | 30 | 27 |
| 21 | 3-Hydroxy-9,10-Dimethoxypterocarpan | 100 | 100 |
| 22 | 5-p-Coumaroylquinic acid | 100 | 100 |
| 23 | adenosine | 100 | 100 |
| 24 | Baohuoside I | 100 | 100 |
| 25 | Danshensu | 100 | 100 |
| 26 | Dihydrotanshinone I | 100 | 100 |
| 27 | tanshinone ΙΙB | 100 | 100 |
| 28 | Tanshinone V | 100 | 100 |
| 29 | Torachrysone 8-O-Glucoside | 100 | 100 |
| 30 | ω-Hydroxyemodin | 100 | 100 |

**Supplementary Date Sheet S3.** The Results of anti-oxidative targets on online database

| **No** | **Search terms** | **Targets in GeneCards** | **Target of "score> 20"** |
| --- | --- | --- | --- |
| 1 | oxidative stress | 9244 | 135 |
| 2 | anti-oxidant | 982 | 0 |

**Supplementary Date Sheet S4.** Potential direct targets and indirect targets of anti-oxidant induced by Huolisu oral liquid

| **No** | **Gene** | **Targets** | **Type** | **Dgree** |
| --- | --- | --- | --- | --- |
| 1 | GAPDH | Glyceraldehyde-3-phosphate dehydrogenase liver | direct targets | 37 |
| 2 | AKT1 | Serine/threonine-protein kinase AKT | direct targets | 34 |
| 3 | TP53 | Cellular tumor antigen p53 | direct targets | 33 |
| 4 | MAPK1 | MAP kinase ERK2 | direct targets | 32 |
| 5 | JUN | Transcription factor AP-1 | direct targets | 31 |
| 6 | CASP3 | Caspase-3 | direct targets | 30 |
| 7 | MAPK8 | Mitogen-activated protein kinase 8 | direct targets | 30 |
| 8 | PTGS2 | Prostaglandin G/H synthase 2 | direct targets | 29 |
| 9 | NOS3 | Nitric-oxide synthase, endothelial | direct targets | 27 |
| 10 | HMOX1 | Heme oxygenase 1 | direct targets | 26 |
| 11 | MPO | Myeloperoxidase | direct targets | 25 |
| 12 | EDN1 | Endothelin-1 | direct targets | 25 |
| 13 | FOS | [Proto-oncogene c-Fos](https://tcmspw.com/target.php?qt=4478) | direct targets | 24 |
| 14 | MAPK14 | Mitogen-activated protein kinase 14 | direct targets | 23 |
| 15 | APP | Beta amyloid A4 protein | direct targets | 23 |
| 16 | HSP90AA1 | Heat shock protein HSP 90-alpha | indirect targets | 23 |
| 17 | ESR1 | Estrogen receptor alpha | direct targets | 22 |
| 18 | NOS2 | [Nitric oxide synthase, inducible](https://tcmspw.com/target.php?qt=3) | direct targets | 21 |
| 19 | GSR | Glutathione reductase | direct targets | 20 |
| 20 | ICAM1 | [Intercellular adhesion molecule 1](https://tcmspw.com/target.php?qt=4287) | direct targets | 20 |
| 21 | CAV1 | Caveolin-1 | indirect targets | 20 |
| 22 | NOS1 | [Nitric-oxide synthase, brain](https://tcmspw.com/target.php?qt=28) | direct targets | 20 |
| 23 | MAP2K1 | Dual specificity mitogen-activated protein kinase kinase 1 | indirect targets | 18 |
| 24 | HSPA5 | 78 kDa glucose-regulated protein | direct targets | 18 |
| 25 | HSPA1A | Heat shock 70 kDa protein 1 | direct targets | 17 |
| 26 | MAPK9 | c-Jun N-terminal kinase 2 | indirect targets | 17 |
| 27 | F2 | Thrombin | direct targets | 16 |
| 28 | MAPK10 | c-Jun N-terminal kinase 3 | direct targets | 16 |
| 29 | PARP1 | Poly [ADP-ribose] polymerase-1 | direct targets | 14 |
| 30 | GSTP1 | [Glutathione S-transferase P](https://tcmspw.com/target.php?qt=733) | direct targets | 12 |
| 31 | REL | [Transcription factor p65](https://tcmspw.com/target.php?qt=4565) | indirect targets | 10 |
| 32 | VCP | Transitional endoplasmic reticulum ATPase | direct targets | 10 |
| 33 | TYR | Tyrosinase | direct targets | 9 |
| 34 | YBX1 | Y-box-binding protein 1 | indirect targets | 8 |
| 35 | HMOX2 | Heme oxygenase 2 | indirect targets | 8 |
| 36 | PTGS1 | [Prostaglandin G/H synthase 1](https://tcmspw.com/target.php?qt=6) | indirect targets | 8 |
| 37 | XDH | Xanthine dehydrogenase | direct targets | 6 |
| 38 | THBD | Thrombomodulin | indirect targets | 6 |
| 39 | DCT | L-dopachrome tautomerase | indirect targets | 5 |
| 40 | CTSA | Lysosomal protective protein | indirect targets | 5 |
| 41 | TYRP1 | 5,6-dihydroxyindole-2-carboxylic acid oxidase | indirect targets | 5 |
| 42 | GSTM1 | [Glutathione S-transferase Mu 1](https://tcmspw.com/target.php?qt=2952) | direct targets | 5 |
| 43 | MAPK8IP3 | C-Jun-amino-terminal kinase-interacting protein 3 | indirect targets | 5 |
| 44 | SERPIND1 | Heparin cofactor 2 | indirect targets | 4 |
| 45 | SLC6A4 | [Sodium-dependent serotonin transporter](https://tcmspw.com/target.php?qt=290) | direct targets | 4 |
| 46 | SCN5A | [Sodium channel protein type 5 subunit alpha](https://tcmspw.com/target.php?qt=70) | direct targets | 3 |
| 47 | ELF3 | ETS-related transcription factor Elf-3 | indirect targets | 3 |
| 48 | WNK1 | Serine/threonine-protein kinase WNK1 | indirect targets | 2 |
| 49 | AOX1 | Aldehyde oxidase | indirect targets | 2 |
| 50 | MAOA | Amine oxidase [flavin-containing] A | direct targets | 2 |
| 51 | VIMP | Selenoprotein S | indirect targets | 2 |
| 52 | NDUFS1 | NADH-ubiquinone oxidoreductase 75 kDa subunit | indirect targets | 2 |
| 53 | PLIN2 | Perilipin-2 | indirect targets | 2 |
| 54 | OXSR1 | Serine/threonine-protein kinase OSR1 | direct targets | 1 |

**Supplementary Date Sheet S5. Molecular docking of target proteins and its reverse selected chemical components**

| **Targets** | **Compounds** | **The binding energies (kcal·mol^-1^)** |
| --- | --- | --- |
| JUN | Kaempferol | -7.8 |
| MAPK1 | ω-Hydroxyemodin | -7.6 |
| AKT1 | 5-p-Coumaroylquinic acid | -7.4 |
| AKT1 | Kaempferol | -7.4 |
| JUN | Formononetin | -7.2 |
| GAPDH | Adenosine | -7.0 |
| JUN | Danshensu | -6.5 |
| MAPK1 | Torachrysone 8-O-Glucoside | -6.2 |
| MAPK1 | Adenosine | -6.1 |
| TP53 | Gallic acid | -5.3 |

**Supplementary Date Sheet S6.** Results of DPPH free radical scavenging experiment

| **Dilution times** | **Sample removal rate/%** |
| --- | --- |
| 400 | 25.84% |
| 200 | 33.43% |
| 100 | 61.10% |
| 50 | 90.31% |
| 20 | 91.29% |

**Supplementary Figure S1.** The mass fragment and fragmentation pathway of some compounds. **(A)** 2"-O-RhamnosylikarisideⅡ, **(B)** Emodin, **(C)** Stilbene glucoside, **(D)** Salvianolic acid C, **(E)** Cryptotanshinone.
